# Clinical detection of human probiotics and human pathogenic bacteria by using a novel high-throughput platform based on next generation sequencing

**DOI:** 10.1186/2043-9113-4-1

**Published:** 2014-01-13

**Authors:** Chih-Min Chiu, Feng-Mao Lin, Tzu-Hao Chang, Wei-Chih Huang, Chao Liang, Ting Yang, Wei-Yun Wu, Tzu-Ling Yang, Shun-Long Weng, Hsien-Da Huang

**Affiliations:** 1 Institute of Bioinformatics and Systems Biology, National Chiao Tung University, Hsin-Chu 300, Taiwan; 2 Graduate Institute of Biomedical Informatics, Taipei Medical University, Taipei, Taiwan; 3 Department of Biological Science and Technology, National Chiao Tung University, Hsin-Chu 300, Taiwan; 4 Department of Obstetrics and Gynecology, Hsinchu Mackay Memorial Hospital, Hsinchu, Taiwan; 5 Mackay Medicine, Nursing and Management College, Taipei, Taiwan; 6 Department of Medicine, Mackay Medical College, New Taipei City, Taiwan

## Abstract

**Background:**

The human body plays host to a vast array of bacteria, found in oral cavities, skin, gastrointestinal tract and the vagina. Some bacteria are harmful while others are beneficial to the host. Despite the availability of many methods to identify bacteria, most of them are only applicable to specific and cultivable bacteria and are also tedious. Based on high throughput sequencing technology, this work derives 16S rRNA sequences of bacteria and analyzes probiotics and pathogens species.

**Results:**

We constructed a database that recorded the species of probiotics and pathogens from literature, along with a modified Smith-Waterman algorithm for assigning the taxonomy of the sequenced 16S rRNA sequences. We also constructed a bacteria disease risk model for seven diseases based on 98 samples. Applicability of the proposed platform is demonstrated by collecting the microbiome in human gut of 13 samples.

**Conclusions:**

The proposed platform provides a relatively easy means of identifying a certain amount of bacteria and their species (including uncultivable pathogens) for clinical microbiology applications. That is, detecting how probiotics and pathogens inhabit humans and how affect their health can significantly contribute to develop a diagnosis and treatment method.

## Background

High throughput sequencing can analyze a large amount of sequences, enabling sequencing of 16S rRNA to identify complex bacteria species of pathogens and probiotic bacteria. Many naturally occurring bacteria form a complex population in the environment. The human body plays host to a vast array of bacteria, found in oral cavities, skin, gastrointestinal tract and the vagina. Some bacteria are harmful while others are beneficial to the host.

A pathogen is a microorganism that causes disease in its host. For example, bacterial pathogen include *Corynebacterium diphtheria* (causes diphtheria), *Listeria monocytogenes* (causes food poisons), and *Legionella pneumophila* (causes Legionnaires’ disease). Probiotics, another microorganism, benefit the host and has received considerable attention in recent years. A FAO report in 2001 [1] cited the advantages of probiotics as increasing immunity [2,3], reducing gastrointestinal discomfort [4,5], and protecting the flora within urogenital tract [6]. As is well known, probiotics can ameliorate symptoms of diseases [7] and reduce the risk of suffering from diseases [8,9].

Despite the availability of many approaches to identify probiotics and pathogens, most of them are only applicable to specific and cultivable bacteria but time consuming. For instance, conventional methods detect growth of cultured bacteria in approximately two days, or an additional five days to obtain no-growth culture results [10], which is laborious. Besides, some bacteria cannot be cultured [11], subsequently increasing the difficulty of specifying pathogenic bacteria. Moreover, it is hard to determine whether an infection is caused by one or more bacteria types.

16S rRNA sequences, capable of identifying bacteria on a molecular level, can detect uncultivable bacteria [12]. Use of 16S rRNA sequencing can overcome some problems of conventional culture method [13]. Although 16S rRNA sequencing is a more effective means of identifying bacteria than conventional culture method, 16S rRNA sequencing takes a considerable amount of time in amplifying DNA sequences [14]. Sanger sequencing known as “first-generation” or “conventional” sequencing has been used for DNA sequencing for almost two decades. Next generation sequencing (NGS) can analyze large-scale sequences quicker, enable massively parallel analysis, reduce reagent costs and the size of sample components, and perform high throughput [15]. Thus NGS is more efficient than the Sanger method, which generates one read per sample. In addition, NGS of 16S rRNA more easily identify cultivable or uncultivable bacteria [12].

Because of the improvement of sequencing technology and Bioinformatics approaches, the accuracy in distinguishing bacteria with those methods has been increased. Based on high throughput sequencing technology, this work identifies 16S rRNA sequences of bacteria and analyzes bacteria species. High-throughput sequencing can sequence a large number of 16S rRNA sequence more efficiently; with high-throughput sequencing, researchers can acquire information to identify pathogens and probiotic bacteria [16-18].

## Results

### Platform application: gut probiotics and pathogens detection

**Figure 1:**
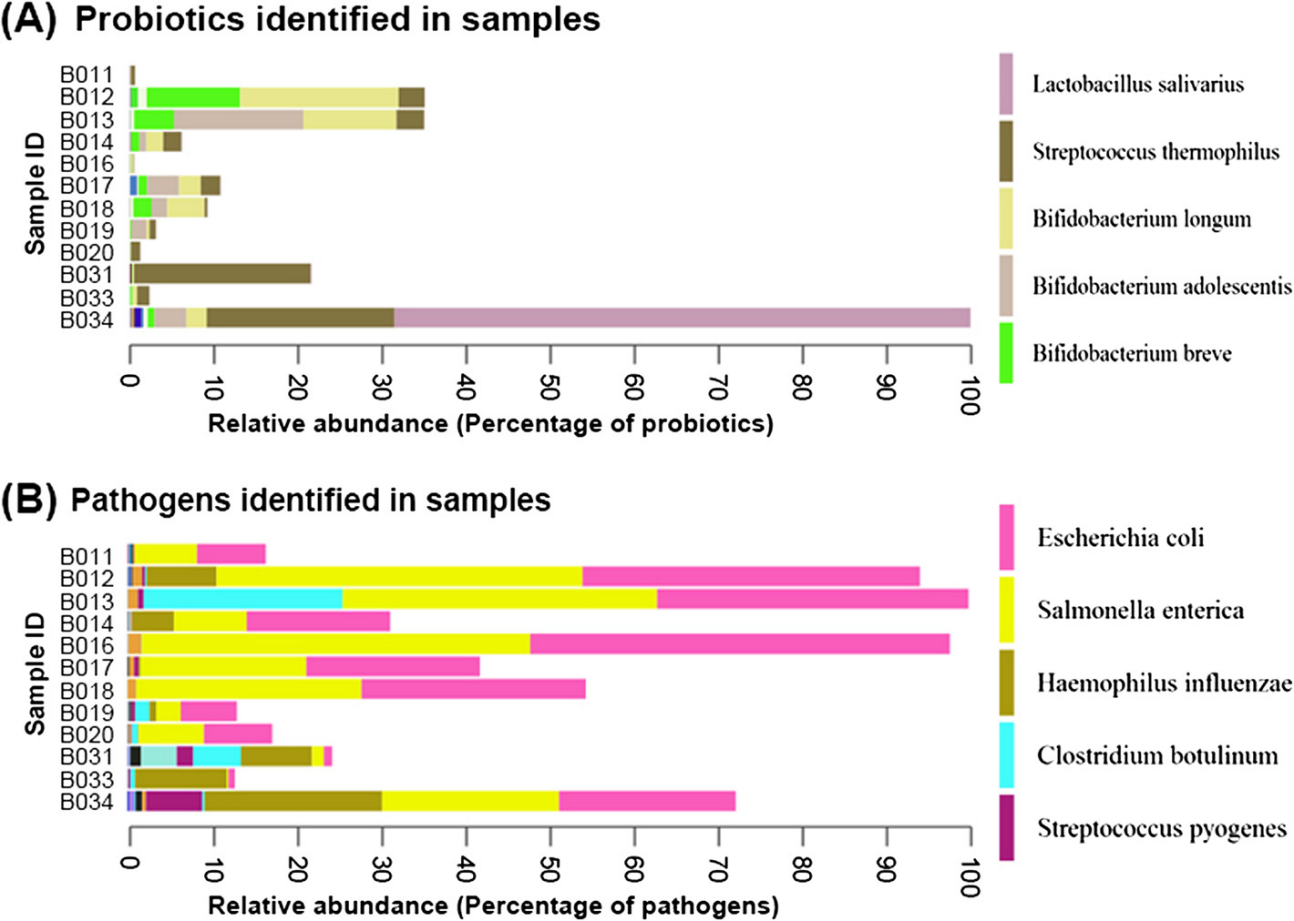
**Relative abundance of probiotics and pathogenic bacteria from human gut of all samples. (A)** The percentage of probiotics was identified in the samples. **(B)** The proportion of pathogenic bacteria was identified in the samples in the case study.

The read statistics of quality filtering and taxonomy assignment are demonstrated in Table 1. Figure 1A illustrated the percentage of probiotics detected by the proposed platform. Table 2 listed the quantities (matched sequenced reads) of probiotics identified in the samples in the case study. The top three identified probiotics in 12 samples are *Lactococcus salivarius*, *Streptococcus thermophilus*, and *Bifidobacterium longum*. Figure 1B and Table 3 listed the proportion and quantities of pathogens, of which top three pathogens are *Escherichia coli, Salmonella enteric*, and *Haemophilus influenza.*

**Table 1:** Results of quality filtering and taxonomy assignment

**ID**	**Raw reads**	**QC**		**Bacteria identified**		**Probiotics**		**Pathogens**	
B011	125420	117451	93.65%	90952	77.44%	60	0.07%	3509	3.86%
B012	132240	120134	90.85%	94679	78.81%	3457	3.65%	20109	21.24%
B013	151876	142585	93.88%	99025	69.45%	3452	3.49%	21341	21.55%
B014	134619	126784	94.18%	95377	75.23%	611	0.64%	6665	6.99%
B016	135457	126507	93.39%	89407	70.67%	49	0.05%	20870	23.34%
B017	141682	131968	93.14%	89465	67.79%	1064	1.19%	8944	10.00%
B018	111228	102382	92.05%	56981	55.66%	910	1.60%	11630	20.41%
B019	128532	120719	93.92%	76877	63.68%	305	0.40%	2775	3.61%
B020	128441	121957	94.95%	89618	73.48%	123	0.14%	3673	4.10%
B031	140941	132311	93.88%	97962	74.04%	2129	2.17%	5194	5.30%
B033	142462	134554	94.45%	80548	59.86%	229	0.28%	2725	3.38%
B034	148854	140059	94.09%	106050	75.72%	9857	9.29%	15436	14.56%
Total	1621752	1517411	93.54%	1066941	70.31%	22246	2.09%	122871	11.52%

**Table 2:** The quantities (matched sequenced reads) of probiotics identified in the samples in the case study

**Probiotics**	**B011**	**B012**	**B013**	**B014**	**B016**	**B017**	**B018**	**B019**	**B020**	**B031**	**B033**	**B034**	
*Bacillus coagulans*	0	81	6	1	2	0	3	0	1	0	9	1	**104**
*Bifidobacterium adolescentis*	4	3	1520	81	1	372	185	177	1	5	0	375	**2724**
*Bifidobacterium animalis*	0	101	37	3	1	16	32	1	1	0	0	50	**242**
*Bifidobacterium bifidum*	0	3	3	0	0	84	2	0	0	0	0	21	**113**
*Bifidobacterium breve*	0	1092	465	96	6	102	212	13	2	9	18	79	**2094**
*Bifidobacterium longum	3	1859	1092	198	27	256	439	34	5	15	55	238	**4221**
*Lactobacillus brevis*	0	0	0	0	0	0	1	0	0	0	0	10	**11**
*Lactobacillus casei*	0	10	1	1	0	0	0	0	0	1	0	0	**13**
*Lactobacillus fermentum*	0	0	0	0	1	0	0	4	0	1	0	28	**34**
*Lactobacillus gasseri*	0	0	0	1	1	0	0	0	0	0	0	77	**79**
*Lactobacillus johnsonii*	0	0	0	0	0	0	0	0	0	0	0	7	**7**
*Lactobacillus paracasei*	0	1	2	0	0	0	0	0	0	0	0	1	**4**
*Lactobacillus plantarum*	1	0	0	2	0	0	0	0	0	0	0	0	**3**
*Lactobacillus reuteri*	0	0	0	1	0	0	0	0	0	0	0	1	**2**
*Lactobacillus rhamnosus*	0	1	0	0	0	0	0	0	0	0	0	2	**3**
*Lactobacillus salivarius	2	1	2	8	1	5	3	1	3	11	2	6753	**6792**
*Lactococcus lactis*	2	0	0	6	1	0	2	0	0	16	1	10	**38**
*Streptococcus thermophilus	48	305	324	213	8	229	31	75	110	2071	144	2204	**5762**
	**60**	**3457**	**3452**	**611**	**49**	**1064**	**910**	**305**	**123**	**2129**	**229**	**9857**	

For each species, if the number of reads is 0 for all samples, that species was not shown.

*The leading three probiotics are *Lactococcus salivarius*, *Streptococcus thermophilus* and *Bifidobacterium longum*.

**Table 3:** The quantities (matched sequenced reads) of pathogens identified in the samples in the case study

**Pathogens**	**B011**	**B012**	**B013**	**B014**	**B016**	**B017**	**B018**	**B019**	**B020**	**B031**	**B033**	**B034**	
*Bordetella pertussis*	0	1	0	0	0	1	0	0	0	0	0	0	**2**
*Brucella abortus*	0	0	0	0	0	0	0	0	0	0	0	0	**0**
*Brucella melitensis*	0	0	0	0	0	0	0	0	0	0	0	0	**0**
*Campylobacter jejuni*	0	0	0	11	0	0	0	0	0	11	1	40	**63**
*Clostridium botulinum*	0	38	5048	4	5	2	1	361	153	1211	115	59	**6997**
*Clostridium difficile*	0	0	1	0	0	0	0	0	0	0	0	0	**1**
*Clostridium perfringens*	0	1	2	0	0	0	0	3	10	24	12	93	**145**
*Corynebacterium diphtheriae*	0	1	0	1	0	0	0	0	0	1	0	0	**3**
*Enterococcus faecalis*	57	13	1	4	8	4	0	20	5	19	6	38	**175**
*Enterococcus faecium*	41	8	2	6	5	2	1	22	3	13	1	32	**136**
*Escherichia coli	1744	8560	7900	3637	10651	4404	5691	1424	1733	210	165	4483	**50602**
*Haemophilus influenzae	2	1771	2	1055	8	49	1	171	15	1802	2322	4502	**11700**
*Neisseria meningitidis*	0	2	0	3	1	0	1	1	1	1	1	1	**12**
*Pseudomonas aeruginosa*	1	6	6	4	2	2	3	1	3	0	0	3	**31**
*Salmonella enterica	1570	9291	7978	1849	9864	4209	5726	622	1658	303	44	4495	**47609**
*Shigella sonnei*	41	243	239	32	308	122	192	8	41	1	1	98	**1326**
*Staphylococcus aureus*	0	0	0	0	0	0	0	0	1	0	0	0	**1**
*Staphylococcus epidermidis*	0	0	0	0	0	0	0	1	1	0	0	0	**2**
*Streptococcus agalactiae*	0	69	3	0	5	0	1	0	0	3	6	5	**92**
*Streptococcus pneumoniae*	46	26	9	16	1	36	3	46	25	272	5	154	**639**
*Streptococcus pyogenes*	7	76	149	14	10	112	9	94	23	417	45	1428	**2384**
*Vibrio cholerae*	0	3	0	0	0	1	1	0	1	0	1	0	**7**
*Yersinia pestis*	0	0	1	29	2	0	0	1	0	906	0	5	**944**
	**3509**	**20109**	**21341**	**6665**	**20870**	**8944**	**11630**	**2775**	**3673**	**5194**	**2725**	**15436**	

For each species, if the number of reads is 0 for all samples, that species was not shown.

*The leading three pathogens are *Escherichia coli, Salmonella enterica* and *Haemophilus influenzae.*

Table 4 listed the results of disease risk evaluations. It showed that three diseases of two samples (B031 and B034) had similar distributions in the control group. The three diseases are obesity, colorectal cancer, and constipation. Sample B031 had reached the significance level with P-value 0.0333 and 0.0121 < 0.05 of distribution in constipation and colorectal cancer respectively compared to 98 samples control group using binomial test. Sample B034 had reached the significance level with P-value 0.00257 and 0.0121 < 0.05 of distribution in obesity and colorectal cancer. Evaluated by the association of bacterial risk markers and disease, the results suggested that these two samples had higher risk than 98 samples control group in constipation, colorectal cancer, and obesity. Their enterotypes of gut probiotics and pathogens may be one of risk factors which would cause disease.

**Table 4:** The result of disease risk evaluations of 12 samples

**Disease**	**B011**	**B012**	**B013**	**B014**	**B016**	**B017**	**B018**	**B019**	**B020**	**B031**	**B033**	**B034**
***Constipation***	2.67E-01	2.67E-01	2.67E-01	1.00E + 00	1.00E + 00	2.67E-01	1.00E + 00	1.00E + 00	2.67E-01	**3.34E-02**	2.67E-01	1.00E + 00
***Obesity***	1.34E-01	1.34E-01	1.00E + 00	1.34E-01	1.00E + 00	1.34E-01	1.34E-01	1.00E + 00	1.00E + 00	1.34E-01	1.00E + 00	**2.57E-03**
***IBS***	3.33E-01	7.06E-01	1.00E + 00	3.33E-01	1.00E + 00	3.33E-01	3.33E-01	1.00E + 00	1.10E-01	1.10E-01	7.06E-01	3.33E-01
***Ulcerative colitis***	9.30E-02	4.15E-01	1.00E + 00	4.15E-01	1.00E + 00	9.30E-02	4.15E-01	1.00E + 00	9.30E-02	1.00E + 00	1.00E + 00	1.00E + 00
***Colorectal cancer***	4.88E-01	2.59E-01	9.35E-01	7.47E-01	7.47E-01	7.47E-01	2.59E-01	4.88E-01	4.88E-01	**1.22E-02**	7.47E-01	**1.22E-02**
***Atopic dermatitis***	1.83E-01	1.00E + 00	1.00E + 00	1.00E + 00	1.00E + 00	1.83E-01	1.83E-01	1.00E + 00	1.00E + 00	1.00E + 00	1.00E + 00	1.83E-01
***Allergic rhinitis***	1.89E-01	1.00E + 00	1.00E + 00	1.00E + 00	1.00E + 00	1.89E-01	1.89E-01	1.89E-01	1.00E + 00	1.89E-01	1.00E + 00	1.89E-01

The bold numbers represent two samples had reached significance level with P-value less than 0.05 of distribution in three diseases compared to 98 sample control group using evaluation model.

### Reproducibility and accuracy evaluation of proposed platform

Two replicated experiments of four samples were performed to estimate the reproducibility of the proposed platform. The results of repeated experiments were consistent. The similarity between two repeated experiments were calculated by using UniFrac [19]. Results of each sample pair (replicate 1 and 2) closely resemble each other. The similarity of UniFrac distance of each sample pair is higher than 0.96 (0.9617 for B014, 0.9872 for B018, 0.9914 for B020, 0.9722 for B033). This implies that the analysis results are reproducible.

Next, accuracy of the platform is evaluated by adding *Lactobacillus reuteri* to a stool sample (B050). Sample B050 contains 24,408 assigned taxons, and *Lactobacillus reuteri* has no detected count. Whether the counts of this species in positive control sample (B050S_L) are elevated must be determined. Analysis results indicate that 27,113 taxons are detected in sample B050S_L. In fact, the detected counts of *Lactobacillus reuteri* in sample B050S_L are 1,430, and the percentage of *Lactobacillus reuteri* markedly increases from 0% to 5%.

In short, our platform is accurate and reproducible in terms of detecting the quantities of bacterial species of the proposed platform. The results evaluate the accuracy and feasibility of proposed platform in order to identify probiotics and pathogens. While requiring only about one day for detection, not limited in identifying certain bacteria, the proposed platform can detect and quantify multiple bacteria simultaneously.

## Discussion

Because of the constraint of costs and technical limitations, 16S rRNA sequences obtained in most databases are partial sequences. Many studies thus assign taxonomy by using partial 16S rRNA sequences. In our probiotics and pathogens 16S rRNA sequence database, 17,964 sequences are collected from NCBI nucleotide database, NCBI 16S microbial rRNA database, Greengenes database, and SILVA. Our probiotics and pathogens 16S rRNA database contain less than 39% of 16S rRNA sequences which are longer than 1400 bps. Only 9% of the sequences are close to full length.

This work extracts the V4 region from full length 16S rRNA of microbiome in the human gut as a platform application. Some 16S rRNA variable regions are more dependable than other regions in assigning taxonomy like V3 and V4 [20,21]; in addition, some 16S rRNA variable regions are much conserved. The proportion and diversity of probiotics and pathogens may be made diverse by using different 16S rRNA variable regions. The proposed platform is also applicable to other 16S rRNA variable regions for taxonomy assignment. Importantly, a more appropriate region than others must be selected to produce an outcome that is close to full length 16S rRNA sequence.

This work further attempt is to collect common probiotics and pathogens from the literature. Although it may be incomplete, recent advances in sequencing technology make it possible to identify and define an increasing number of bacteria, implying an obvious increase in the number of identified probiotics and pathogens in the future. Efforts are underway in our laboratory to update the list of used probiotics and pathogens.

Previous studies [22-24] identified pathogen or probiotic bacteria by using antibody, 16S rRNA gene microarrays, fluorescence *in situ* hybridization (FISH), and proteomic methods. In this work, the proposed platform can detect various pathogens and probiotics based on 16S rRNA (rDNA) sequences of bacteria using NGS and Bioinformatics method. An average of 126,451 reads was acquired per sample in this work. It is doubt that the sequencing depth is enough to detect a small amount of probiotics and pathogens. Although increasing the coverage of sequencing can advance the sensitivity of detecting probiotics and pathogens, the sequencing cost will increase. It is important to work out an appropriate coverage of sequencing for detecting probiotics and pathogens.

The results of disease risk evaluations revealed that most of 12 samples did not have resembled distributions of bacteria markers with control group. Only two samples had reached the significance level of distributions. The reason for the phenomenon may be the overlapped bacteria markers between diseases. 28 markers are used in colorectal cancer, and 17 markers are used in irritable bowel syndrome. Six markers are overlapped. For sample B031, the significant distributions in colorectal cancer were partly contributed to the significance in irritable bowel syndrome owing to the overlapped markers. Similarly, two overlapped markers for sample B034 were in colorectal cancer and obesity. In this kind of speculation, the influence of colorectal cancer to irritable bowel syndrome would be six (overlapped markers of CC and IBS) over seventeen (markers of IBS), and the influence of colorectal cancer to obesity would be two (overlapped markers of CC and obesity) over nine (markers of obesity). In addition, the influence of colorectal cancer to constipation and ulcerative colitis would be one over six and two over ten, respectively.

In addition to that some bacteria markers in species level are belong to the marker of genus level and species level, both genus marker and species markers may have associated with affecting the distributions mutually. Continually, collecting more markers and evaluating the distributions with markers in the same level are required for constructing a global prediction model in Taiwanese.

## Conclusions

This work constructed a bacterial disease risk evaluation model for seven diseases and developed a novel platform by using NGS and Bioinformatics approach. Compared with the traditional bacteria culture method, our proposed platform can reduce experiment time. Besides, the proportion of probiotics and pathogens (including uncultivable pathogens) in the human body can be detected rapidly with 16 s RNA database of probiotics and pathogens. Furthermore, the proposed platform provides further insight into the cause of disease based on the relation of probiotics, pathogens, and disease. For instance, the type of antibiotics can be adjusted if the pathogens of disease are identified from infected patients. In addition, the proposed platform allows researchers to determine whether the intake of probiotics impacts the human body [25-29]. In the future, this preliminary study will be continuously extended for more bacterial disease markers. For more comprehensive applications, this work will also collect bacteria from other parts of human body as control group data. In fact, a detective method of how the probiotics and pathogens inhabit human can provide new insight for human health. It could improve diagnosis and treatment method.

## Methods

**Figure 2:**
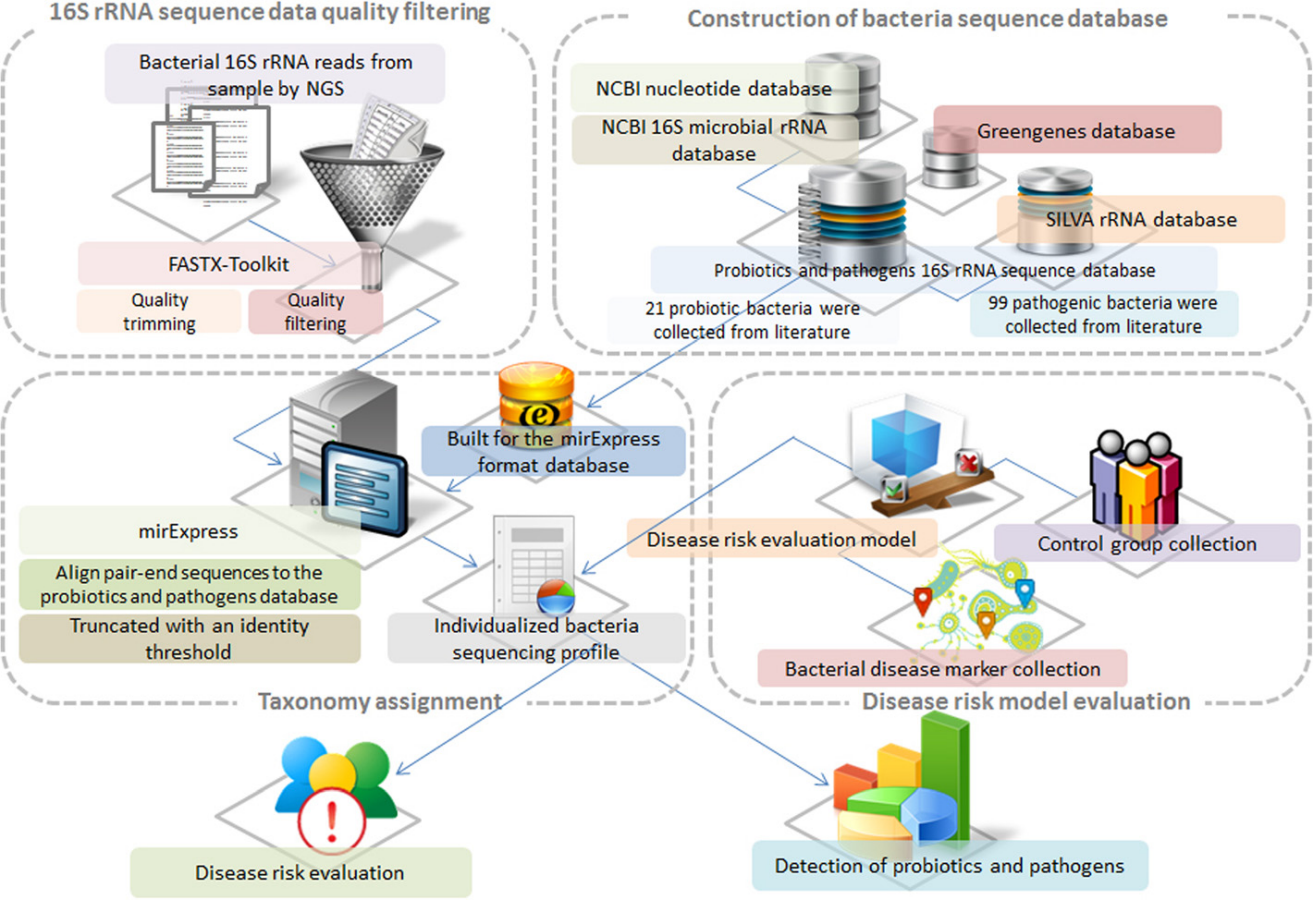
**System flow of bioinformatics analysis in the proposed platform.** The proposed platform comprises the analysis pipeline of NGS, construction of probiotics and pathogens database, bacterial disease risk model evaluation and the application of individualized bacteria sequencing profile.

Figure 2 illustrates the bioinformatics system flow of the proposed platform, which includes analysis pipeline of NGS. The Figure 2 contains four parts: sequence quality filtering, construction of bacteria sequence database, taxonomy assignment, and disease risk model evaluation. The detailed components in the proposed platform are described below.

### Sample collection

In this study, stool samples of 98 Taiwan volunteers were gathered. The samples were collected by Sigma-transwab (Medical Wire) into a tube with Liquid Amies Transport Medium, and stored at 4°C until processing.

### DNA extraction

In the case study, fresh faeces were obtained from participants. DNA was extracted directly on stool samples by using a QIAamp DNA Stool Mini Kit (Qiagen). A swab was vortexed vigorously and incubated at room temperature for 1 min. The sample was then transferred to microcentrifuge tubes containing 560 μl Buffer ASL, vortexed, and incubated at 37°C for 30 min. In addition, the suspension was incubated at 95°C for 15 min, vortexed, and centrifuged at 14,000 rpm for 1 min into pellet stool particles. Extraction was performed following the protocol of the QIAamp DNA Stool Mini Kit. The DNA was eluted with 50 μl Buffer AE, and centrifuged at 14,000 rpm for 1 min. Moreover, the DNA extract was stored at-20°C until further analysis. Finally, DNA extraction was performed, depending on the sample collected.

### Library construction and sequencing for V4 region of 16S ribosomal DNA

The PCR primers, F515 (5′-GTGCCAGCMGCCGCGGTAA-3′) and R806 (5′-GGACTACHVGGGTWTCTAAT-3′), were designed to amplify the V4 domain of bacterial 16S ribosomal DNA as described previously [30]. PCR amplification was performed in a 50 μl reaction volume containing 25 μl 2X Taq Master Mix (Thermo Scientific), 0.2 μM of each forward and reverse primer, and 20 ng DNA template. The reaction conditions consisted of an initial 95°C for 5 min, followed by 30 cycles of 95°C for 30 sec, 54°C for 1 min, and 72°C for 1 min, as well as a final extension of 72°C for 5 min. Next, amplified products were checked by 2% agarose gel electrophoresis and ethidium bromide staining. Amplicons were purified using the AMPure XP PCR Purification Kit (Agencourt), and quantified using Qubit dsDNA HS Assay Kit (Qubit) on Qubit 2.0 Fluorometer (Qubit)–all according to respective manufacturer instructions. For V4 library preparation, Illumina adapters were attached to the amplicons using the Illumina TruSeq DNA Sample Preparation v2 Kit. Purified libraries were applied for cluster generation and sequencing on the MiSeq system. The raw sequence files are available for download at http://clinic.mbc.nctu.edu.tw/.

### 16S rRNA (rDNA) sequence data quality filtering

The raw fastq files obtained by Illumina sequencing machine were quality-filtered using the FASTX-Toolkit^a^. The paired-end 150 bp reads were performed using the minimum acceptable phred quality score of 20, as well as the 70% of bases that must exceed 20 phred quality score. Sequence shorter than 100 nucleotides would be omitted after quality trimming from reads tail. Notably, reads containing ambiguous characters were discarded.

### Construction of probiotics and pathogens database

The list of probiotics and pathogens were obtained from literatures or the claims of official departments. Additional file 1: Table S1 lists species of probiotics which were adapted from both literatures [7,9] and the claims of official departments, such as Taiwan Food and Drug Administration [31] and Health Canada [32]. 99 bacterial pathogens were collected from literature [25,26,33-42] and Taiwan Food and Drug Administration [31] (Additional file 1: Table S2).

The 16S rRNA sequences of probiotics and pathogens used for taxonomy mapping were retrieved from the NCBI nucleotide database, NCBI 16S microbial rRNA database, Greengenes database [43] and SILVA [44]. Following sequence data collection, we assemble partial sequences which used the same species classification and removed redundant sequences. Additionally, we also removed the unique sequence from only one research support with 3% similarity which shared the same species classification with other sequence.

### Taxonomy mapping

To generate taxonomy assignments, the proposed platform invoked a modified Smith-Waterman algorithm from miRExpress [45], which can compare pairs of sequences in parallel, for mapping reads to taxons. miRExpress was designed for identifying the best similarity between sequencing reads and miRNA precursor sequences. In our model, it was modified for identifying multiple hits of 16S rRNA sequence mapping results with similarity threshold 0.97. In order to reduce the storage space of output, the SAM format [46] was used to replace the original miRExpress output format for storing alignment results. Furthermore, two kinds of output format were designed. One format records whole mapped sequencing reads based on taxons. The other one records which taxons could be assigned based on sequencing reads. These two kinds of output could support the important information for assigning sequencing reads to suitable taxon. miRExpress was originally designed for dealing with single-end sequencing data. Therefore, the additional program was added for processing paired-end sequencing data. In this part, both end sequencing reads need to be assigned to the same taxon. If paired-end sequencing reads were mapped to different taxons, this paired sequence would be dropped. The probiotics and pathogens 16S rRNA sequence from our database were built in FASTA format. Following quality filtering, all paired-end sequences were aligned to the probiotics and pathogens database with whole read aligned from one end to the other end. Reads were then truncated with an identity lower than 97%, according to previous research in order to achieve a better compromise between sequences from PCR sequencing errors and taxonomic relatedness [27].

### The construction of Bacterial disease risk evaluation model (BDREM)

To study the associations between bacteria and diseases, we collected related information from literatures. We concerned bacteria that are associated with seven diseases: constipation [28,47,48], obesity [29,49-52], irritable bowel syndrome (IBS) [28,53-58], ulcerative colitis (UC) [53,59-61], colon cancer (CC) [62-64], Atopic Dermatitis (AD) and Allergic rhinitis (AR), were collected positive correlation and negative correlation data, and the individual risk of disease was evaluated.

The association data were majorly collected from case–control studies which the quantities of bacteria were obtained from NGS data, and few well-known bacteria validated by multiple studies through cultural experiments were also included. We further eliminated some conflicted data with both positive and negative correlation between bacteria and disease in different studies.

Health Asians stool samples of 98 Taiwan volunteers were gathered. Following deep sequencing and sequencing data processing, the proportion of 78 bacteria from control group was applied as risk markers (constipation: 6, obesity: 9, IBS: 17, UC: 10, CC: 28, AD: 4, AR: 4) to predict disease risk to seven diseases in this study (Table 5).

**Table 5:** Disease-related biomarkers of seven diseases

**Disease**	**Marker**	**Correlation**	**Lower bound**	**Upper bound**	**Case**	**Control**	**Pubmed ID**
**Constipation**	*Escherichia coli*	-	2.86E-03	1.52E-01	35	35	20039451
	*Roseburia*	-	1.41E-03	4.61E-02	14	12	22315951
	*Lactobacillus*	-	6.10E-05	9.45E-03	14	12	22315951
	*Bifidobacterium*	-	5.39E-05	1.73E-02	14	12	22315951
	*Enterobacteriaceae*	+	1.00E-02	4.26E-01	14	12	22315951
	*Ruminococcus bromii*	+	1.16E-05	4.98E-03	8	15	20014457
**Obesity**	*Prevotella*	-	2.46E-03	5.36E-01	23	13	20876719
	*Bifidobacterium*	-	5.39E-05	1.73E-02	33	30	19498350
	*Lachnospiraceae*	-	3.11E-03	6.74E-02	3	3	19164560
	*Verrucomicrobiae*	-	1.43E-05	1.78E-02	3	3	19164560
	*Akkermansia*	-	1.43E-05	1.78E-02	3	3	19164560
	*Faecalibacterium prausnitzii*	+	7.70E-04	2.15E-02	15	13	19849869
	*Lactobacillus*	+	6.10E-05	9.45E-03	20	20	19774074
	*Coriobacteriaceae*	+	3.26E-05	4.72E-03	3	3	19164560
	*Erysipelotrichaceae*	+	1.35E-04	6.64E-03	3	3	19164560
**Ulcerative colitis**	*Bacteroides uniformis*	-	7.63E-04	5.44E-02	13	22	21073731
	*Bacteroides vulgatus*	-	1.55E-03	4.21E-02	13	22	21073731
	*Parabacteroides distasonis*	-	2.22E-05	1.68E-03	13	22	21073731
	*Faecalibacterium prausnitzii*	-	7.70E-04	2.15E-02	13	27	19235886
	*Firmicutes*	-	9.18E-02	4.50E-01	13	27	19235886
	*Clostridium*	-	2.48E-03	6.03E-02	31	30	21253779
	*Clostridium leptum*	-	9.65E-06	1.05E-03	13	27	19235886
	*Bifidobacterium*	-	5.39E-05	1.73E-02	13	27	19235886
	*Bacteroides ovatus*	-	2.04E-04	1.81E-02	13	22	21073731
	*Escherichia coli*	+	2.86E-03	1.52E-01	9	9	16954244
**Atopic dermatitis**	*Lactobacillus*	-	6.10E-05	9.45E-03	68	256	17604093
	*Bifidobacteriales*	-	8.09E-05	1.84E-02	7	27	20626364
	*Bacteroides*	+	6.56E-02	6.37E-01	68	256	17604093
	*Clostridium perfringens*	+	0.00E + 00	1.06E-04	15	15	21963389
**Colorectal cancer**	*Bacteroides uniformis*	-	7.63E-04	5.44E-02	46	56	21850056
	*Roseburia*	-	1.41E-03	4.61E-02	46	56	21850056
	*Fusobacterium*	-	3.32E-05	2.64E-02	50	38	7574628
	*Eubacterium*	-	1.36E-03	7.92E-02	46	56	21850056
	*Coprococcus*	-	1.91E-05	2.89E-03	21	23	20740058
	*Collinsella aerofaciens*	-	2.39E-05	2.09E-03	50	38	7574628
	*Alistipes*	-	4.07E-04	2.60E-02	46	56	21850056
	*Sutterellaceae*	-	9.39E-04	4.85E-02	46	56	21850056
	*Escherichia*	+	3.05E-03	1.85E-01	46	56	21850056
	*Shigella*	+	1.51E-03	8.84E-02	46	56	21850056
	*Bacteroides fragilis*	+	7.22E-06	1.92E-02	46	56	21850056
	*Porphyromonas*	+	0.00E + 00	1.59E-05	46	56	21850056
	*Faecalibacterium prausnitzii*	+	7.70E-04	2.15E-02	50	38	7574628
	*Ruminococcus albus*	+	0.00E + 00	4.95E-04	50	38	7574628
	*Streptococcus*	+	1.12E-04	6.83E-03	46	56	21850056
	*Blautia hansenii*	+	0.00E + 00	6.77E-05	50	38	7574628
	*Enterococcus*	+	0.00E + 00	1.19E-04	46	56	21850056
	*Bifidobacterium angulatum*	+	0.00E + 00	2.60E-05	50	38	7574628
	*Blautia producta*	+	0.00E + 00	1.10E-04	50	38	7574628
	*Ruminococcus gnavus*	+	1.04E-05	2.64E-03	50	38	7574628
	*Eubacterium eligens*	+	7.09E-05	2.05E-02	50	38	7574628
	*Eubacterium rectale*	+	8.13E-05	1.34E-02	50	38	7574628
	*Bacteroides stercoris*	+	4.87E-05	2.94E-02	50	38	7574628
	*Enterobacteriales*	+	1.00E-02	4.26E-01	10	10	21647227
	*Erysipelotrichaceae*	+	1.35E-04	6.64E-03	50	38	7574628
	*Dorea*	+	5.67E-05	6.08E-03	21	23	20740058
	*Bifidobacterium longum*	+	1.56E-05	3.60E-03	50	38	7574628
	*Faecalibacterium*	+	1.66E-03	6.79E-02	21	23	20740058
**Irritable bowel syndrome**	*Bacteroides uniformis*	-	7.63E-04	5.44E-02	11	22	21073731
	*Bacteroides vulgatus*	-	1.55E-03	4.21E-02	11	22	21073731
	*Parabacteroides distasonis*	-	2.22E-05	1.68E-03	11	22	21073731
	*Faecalibacterium prausnitzii*	-	7.70E-04	2.15E-02	23	23	22339879
	*Bacteroidetes*	-	2.87E-01	7.95E-01	62	46	21820992
	*Bifidobacterium*	-	5.39E-05	1.73E-02	62	46	21820992
	*Bacteroides ovatus*	-	2.04E-04	1.81E-02	11	22	21073731
	*Faecalibacterium*	-	1.66E-03	6.79E-02	62	46	21820992
	*Escherichia coli*	+	2.86E-03	1.52E-01	14	18	22356587
	*Haemophilus*	+	1.02E-05	1.69E-03	22	22	21741921
	*Fusobacterium*	+	3.32E-05	2.64E-02	23	23	22339879
	*Gammaproteobacteria*	+	1.75E-02	4.69E-01	22	22	21741921
	*Ruminococcus*	+	1.22E-03	4.08E-02	62	46	21820992
	*Enterococcus*	+	0.00E + 00	1.19E-04	23	23	22339879
	*Veillonella*	+	1.12E-05	7.82E-03	26	26	19903265
	*Lactobacillaceae*	+	6.10E-05	9.45E-03	23	23	22339879
	*Dorea*	+	5.67E-05	6.08E-03	62	46	21820992
**Allergic rhinitis**	*Lactobacillus*	-	6.10E-05	9.45E-03	12	12	19714856
	*Bifidobacterium*	-	5.39E-05	1.73E-02	67	20	101
	*Bacteroides fragilis*	+	7.22E-06	1.92E-02	22	22	17893165
	*Faecalibacterium prausnitzii*	+	7.70E-04	2.15E-02	22	22	17893165

The associations between bacterium and disease are majorly collected from case–control studies which the quantities of bacterium are obtained from deep sequencing data. The proportion of 78 bacteria from control group was applied as risk markers (constipation: 6, obesity: 9, IBS: 17, UC: 10, CC: 28, AD: 4, AR: 4) to predict disease risk to seven diseases in this study.

The mathematical formula of BDREM in this study was developed as the following steps. Let *λ* be a *N* × *S* matrix, where *N* is the number of markers selected in the prediction model of constipation and *S* is the number of health subjects in 7 prediction models. *T*_*i*_ was defined as one of the two notches of median for each row of *λ*[65]. *T*_*i*_ is a threshold to distinguish *λ*_*ij*_ from normal proportion level to abnormal (fail to success in one trail of binomial distribution). Smaller notch was selected to *T*_*i*_ when *each* marker was recorded as a negative association to the disease, and a success trail was identified when *λ*_*ij*_ is smaller than *T*_*i*_. On the opposite, larger notch was selected when association was positive, and a success trail was identified when *λ*_*ij*_ is larger than *T*_*i*_.

$$\mathrm{Ti}=\left\{\begin{array}{ll} \text{Medain}\;\text{of}\; & \left\{\lambda_{i1},\lambda_{i2},\ldots ,\lambda_{\mathrm{iS}}\right\}+\frac{1.58\times \text{IQR}}{\sqrt{S}}\;,\\ & \text{positive}\;\text{association}\;\\ \;\text{Medain}\;\text{of}\; & \left\{\lambda_{i1},\lambda_{i2},\ldots ,\lambda_{\mathrm{iS}}\right\}-\frac{1.58\times \text{IQR}}{\sqrt{S}}\;,\\ & \text{negative}\;\text{association}\; \end{array}\right)$$

*Let P*_*j*_ be the probability of successful trails in the *j*^*th*^ column of *λ*. The meaning of *P*_*j*_ is the personal probability that abnormal proportion level happened.

$$P_{j}=\frac{\#\text{success}\;\text{trails}\;\text{in}\;\text{the}\;\text{jth}\;\text{column}\;\text{of}\;\lambda .}{N}$$

*Let P*_*h*_ be the mean of *P*_*j*_. It represents how frequent the abnormal proportion level happened to all *P*_*j*_ in average, regarded as the hypothesized probability of success in each *P*_*j*_.

$$P_{h}=\frac{\sum_{j=1}^{S}P_{j}}{N}$$

Assume *P*_*j*_ obey a binomial distribution, and let *P*_*h*_ be the hypothesized probability (0.05051 for constipation, 0.07239 for obesity, 0.06952 for IBS, 0.05227 for UC, 0.09280 for CC, 0.04924 for AD, 0.05114 for AR). A binomial test was used to *P*_*j*_ and *P*_*h*_. Alpha = 0.05 was choose to judge if a subject is significantly differently from the others in *λ*.

**Figure 3:**
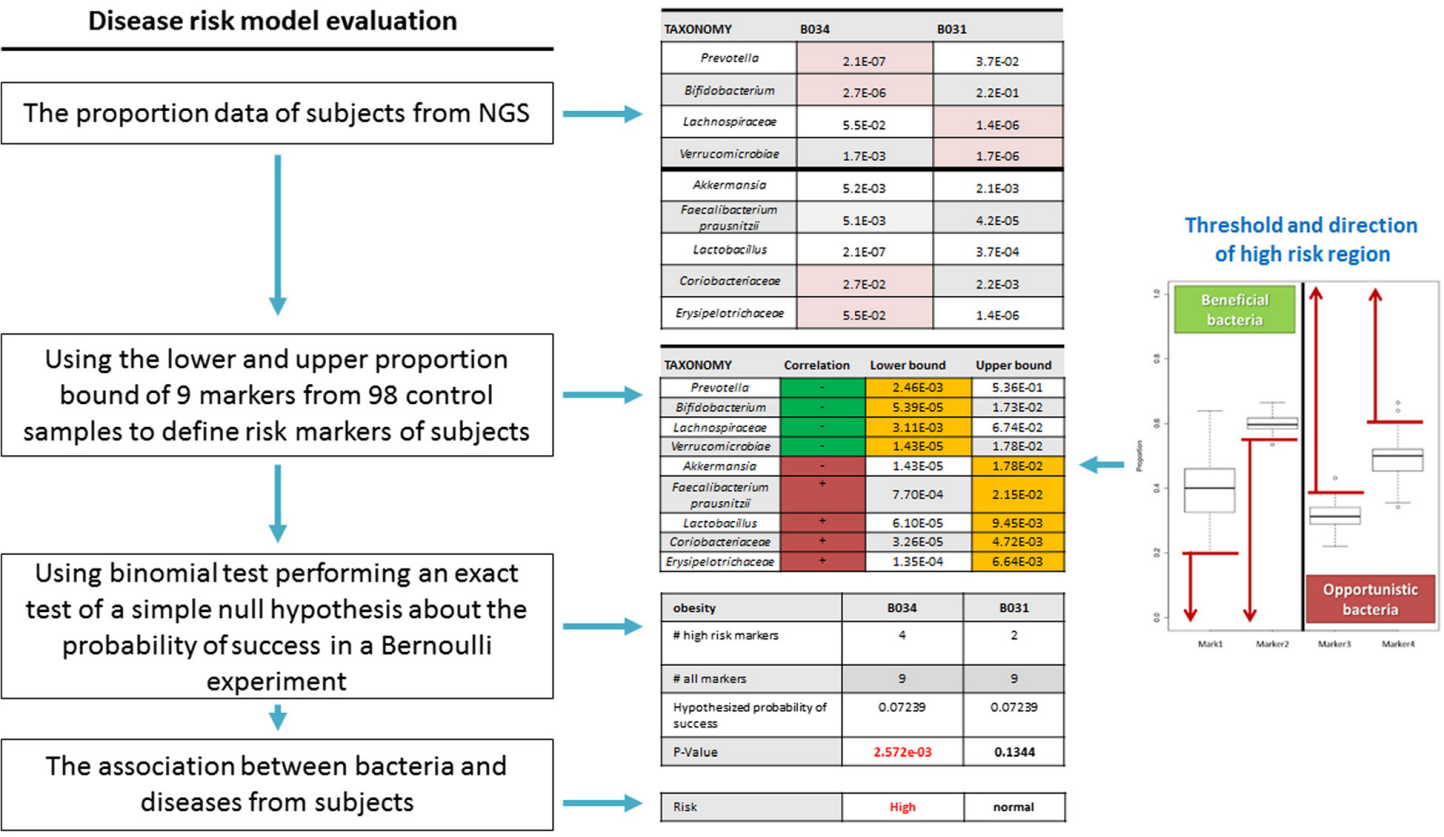
**An example for evaluating the risk of obesity by using bacterial disease risk evaluation model.** The model used lower and upper proportion bound of 9 markers from 98 control samples to define risk markers of these two samples (B034 and B031) following by using binomial test.

Figure 3 illustrated an example for evaluating the risk of obesity of B034 and B031. The model used lower and upper proportion bound of 9 markers from 98 control samples to define risk markers of these two samples following by using binomial test. Four markers of B034 exceed the lower bound and upper bound of obesity. The binomial test P-Value of B034 is 2.572e-03 < 0.05, Since P-Value < = hypothesized probability 0.07239, this case is specifically associated (significantly) with disease than random chance. There are two markers of B031 exceed lower bound of obesity. The P-Value of B031 is 0.1344 > 0.05, the case is no more associated with disease than random chance. As the results, we can assume that B034 had higher probability to cause Obesity.

## Endnote

^a^http://hannonlab.cshl.edu/fastx_toolkit/index.html.

## Competing interests

The authors declare no competing interests.

## Authors’ contributions

HDH conceived and supervised the study. CMC were responsible for the design, computational analyses, implementation of the system, and drafting the manuscript. FML, THC, CL, TY, TLY, SLW, WCH and WYW were in charge of manuscript revision and data update. All authors read and approved the final manuscript.

## Supplementary Material

Additional file 1 **The list of probiotics and pathogens were obtained from literatures or the claims of official departments: Table S1.** The reference list of probiotics. **Table S2.** The reference list of pathogens. Click here for file

## References

[B1] FAO/WHO Health and nutritional properties of probiotics in food including powder milk with live lactic acid bacteria Book Health and nutritional properties of probiotics in food including powder milk with live lactic acid bacteriahttp://www.who.int/foodsafety/publications/fs_management/en/probiotics.pdf. City; 2001

[B2] Kalliomaki M Salminen S Arvilommi H Kero P Koskinen P Isolauri E Probiotics in primary prevention of atopic disease: a randomised placebo-controlled trial Lancet 2001 357 1076 1079 10.1016/S0140-6736(00)04259-8 11297958

[B3] Isolauri E Sutas Y Kankaanpaa P Arvilommi H Salminen S Probiotics: effects on immunity Am J Clin Nutr 2001 73 444S 450S 11157355 10.1093/ajcn/73.2.444s

[B4] Gopal PK Prasad J Smart J Gill HS In vitro adherence properties of lactobacillus rhamnosus DR20 and bifidobacterium lactis DR10 strains and their antagonistic activity against an enterotoxigenic Escherichia coli Int J Food Microbiol 2001 67 207 216 10.1016/S0168-1605(01)00440-8 11518430

[B5] Ogawa M Shimizu K Nomoto K Takahashi M Watanuki M Tanaka R Tanaka T Hamabata T Yamasaki S Takeda Y Protective effect of Lactobacillus casei strain Shirota on Shiga toxin-producing Escherichia coli O157:H7 infection in infant rabbits Infect Immun 2001 69 1101 1108 10.1128/IAI.69.2.1101-1108.2001 11160007 PMC97991

[B6] Reid G Probiotic agents to protect the urogenital tract against infection Am J Clin Nutr 2001 73 437S 443S 11157354 10.1093/ajcn/73.2.437s

[B7] Wan YM Zhu YQ Xia B Luo J Treating TNBS-induced colitis in rats with probiotics Turk J Gastroenterol 2011 22 486 493 22234755 10.4318/tjg.2011.0247

[B8] Taipale T Pienihakkinen K Isolauri E Larsen C Brockmann E Alanen P Jokela J Soderling E Bifidobacterium animalis subsp. lactis BB-12 in reducing the risk of infections in infancy Br J Nutr 2011 105 409 416 10.1017/S0007114510003685 20863419

[B9] Wickens K Black PN Stanley TV Mitchell E Fitzharris P Tannock GW Purdie G Crane J A differential effect of 2 probiotics in the prevention of eczema and atopy: a double-blind, randomized, placebo-controlled trial J Allergy Clin Immunol 2008 122 788 794 10.1016/j.jaci.2008.07.011 18762327

[B10] Jordan JA Durso MB Real-time polymerase chain reaction for detecting bacterial DNA directly from blood of neonates being evaluated for sepsis J Mol Diagn 2005 7 575 581 10.1016/S1525-1578(10)60590-9 16258155 PMC1867550

[B11] Wilson M Bacteriology of Humans: An Ecological Perspective 2008 Hoboken: Wiley-Blackwell

[B12] Goldenberger D Kunzli A Vogt P Zbinden R Altwegg M Molecular diagnosis of bacterial endocarditis by broad-range PCR amplification and direct sequencing J Clin Microbiol 1997 35 2733 2739 9350723 10.1128/jcm.35.11.2733-2739.1997 PMC230051

[B13] Woo PC Lau SK Woo GK Fung AM Ngan AH Hui WT Yuen KY Seronegative bacteremic melioidosis caused by Burkholderia pseudomallei with ambiguous biochemical profile: clinical importance of accurate identification by 16S rRNA gene and groEL gene sequencing J Clin Microbiol 2003 41 3973 3977 10.1128/JCM.41.8.3973-3977.2003 12904433 PMC179777

[B14] Fox GE Magrum LJ Balch WE Wolfe RS Woese CR Classification of methanogenic bacteria by 16S ribosomal RNA characterization Proc Natl Acad Sci U S A 1977 74 4537 4541 10.1073/pnas.74.10.4537 16592452 PMC431980

[B15] Metzker ML Sequencing technologies–the next generation Nat Rev Genet 2010 11 31 46 10.1038/nrg2626 19997069

[B16] Kuczynski J Lauber CL Walters WA Parfrey LW Clemente JC Gevers D Knight R Experimental and analytical tools for studying the human microbiome Nat Rev Genet 2012 13 47 58 22179717 10.1038/nrg3129 PMC5119550

[B17] Woo PC Lau SK Teng JL Tse H Yuen KY Then and now: use of 16S rDNA gene sequencing for bacterial identification and discovery of novel bacteria in clinical microbiology laboratories Clin Microbiol Infect 2008 14 908 934 10.1111/j.1469-0691.2008.02070.x 18828852

[B18] Lazarevic V Whiteson K Huse S Hernandez D Farinelli L Osteras M Schrenzel J Francois P Metagenomic study of the oral microbiota by Illumina high-throughput sequencing J Microbiol Methods 2009 79 266 271 10.1016/j.mimet.2009.09.012 19796657 PMC3568755

[B19] Lozupone C Hamady M Knight R UniFrac–an online tool for comparing microbial community diversity in a phylogenetic context BMC Bioinformatics 2006 7 371 10.1186/1471-2105-7-371 16893466 PMC1564154

[B20] Bokulich NA Joseph CM Allen G Benson AK Mills DA Next-generation sequencing reveals significant bacterial diversity of botrytized wine PloS one 2012 7 e36357 10.1371/journal.pone.0036357 22563494 PMC3341366

[B21] Claesson MJ Wang Q O’Sullivan O Greene-Diniz R Cole JR Ross RP O’Toole PW Comparison of two next-generation sequencing technologies for resolving highly complex microbiota composition using tandem variable 16S rRNA gene regions Nucleic Acids Res 2010 38 e200 10.1093/nar/gkq873 20880993 PMC3001100

[B22] Russmann H Kempf VA Koletzko S Heesemann J Autenrieth IB Comparison of fluorescent in situ hybridization and conventional culturing for detection of Helicobacter pylori in gastric biopsy specimens J Clin Microbiol 2001 39 304 308 10.1128/JCM.39.1.304-308.2001 11136788 PMC87719

[B23] Barber JJ Grichting WL Australia’s media campaign against drug abuse Int J Addict 1990 25 693 708 2265870 10.3109/10826089009061328

[B24] Rigsbee L Agans R Foy BD Paliy O Optimizing the analysis of human intestinal microbiota with phylogenetic microarray FEMS Microbiol Ecol 2011 75 332 342 10.1111/j.1574-6941.2010.01009.x 21155851 PMC3077101

[B25] Maley MW Kociuba K Chan RC Prevention of laboratory-acquired brucellosis: significant side effects of prophylaxis Clin Infect Dis 2006 42 433 434 10.1086/499112 16392095

[B26] Mantur BG Amarnath SK Shinde RS Review of clinical and laboratory features of human brucellosis Indian J Med Microbiol 2007 25 188 202 10.4103/0255-0857.34758 17901634

[B27] Hummelen R Fernandes AD Macklaim JM Dickson RJ Changalucha J Gloor GB Reid G Deep sequencing of the vaginal microbiota of women with HIV PloS one 2010 5 e12078 10.1371/journal.pone.0012078 20711427 PMC2920804

[B28] Lyra A Rinttila T Nikkila J Krogius-Kurikka L Kajander K Malinen E Matto J Makela L Palva A Diarrhoea-predominant irritable bowel syndrome distinguishable by 16S rRNA gene phylotype quantification World J Gastroenterol 2009 15 5936 5945 10.3748/wjg.15.5936 20014457 PMC2795180

[B29] Furet JP Kong LC Tap J Poitou C Basdevant A Bouillot JL Mariat D Corthier G Dore J Henegar C Differential adaptation of human gut microbiota to bariatric surgery-induced weight loss: links with metabolic and low-grade inflammation markers Diabetes 2010 59 3049 3057 10.2337/db10-0253 20876719 PMC2992765

[B30] Caporaso JG Lauber CL Walters WA Berg-Lyons D Lozupone CA Turnbaugh PJ Fierer N Knight R Global patterns of 16S rRNA diversity at a depth of millions of sequences per sample Proc Natl Acad Sci U S A 2011 108 Suppl 1 4516 4522 20534432 10.1073/pnas.1000080107 PMC3063599

[B31] Khamis A Raoult D La Scola B Comparison between rpoB and 16S rRNA gene sequencing for molecular identification of 168 clinical isolates of corynebacterium J Clin Microbiol 2005 43 1934 1936 10.1128/JCM.43.4.1934-1936.2005 15815024 PMC1081344

[B32] Lin J Effect of antibiotic growth promoters on intestinal microbiota in food animals: a novel model for studying the relationship between gut microbiota and human obesity? Front Microbiol 2011 2 53 21833309 10.3389/fmicb.2011.00053 PMC3153029

[B33] Hegerle N Guiso N Epidemiology of whooping cough and typing of Bordetella pertussis Future Microbiol 2013 8 11 1391 1403 10.2217/fmb.13.111 24199799

[B34] Seleem MN Boyle SM Sriranganathan N Brucella: a pathogen without classic virulence genes Vet Microbiol 2008 129 1 14 10.1016/j.vetmic.2007.11.023 18226477

[B35] Zhan P Suo LJ Qian Q Shen XK Qiu LX Yu LK Song Y Chlamydia pneumoniae infection and lung cancer risk: a meta-analysis Eur J Cancer 2011 47 742 747 10.1016/j.ejca.2010.11.003 21194924

[B36] Parkhill J Wren BW Mungall K Ketley JM Churcher C Basham D Chillingworth T Davies RM Feltwell T Holroyd S The genome sequence of the food-borne pathogen campylobacter jejuni reveals hypervariable sequences Nature 2000 403 665 668 10.1038/35001088 10688204

[B37] Guidoboni M Ferreri AJ Ponzoni M Doglioni C Dolcetti R Infectious agents in mucosa-associated lymphoid tissue-type lymphomas: pathogenic role and therapeutic perspectives Clin Lymphoma Myeloma 2006 6 289 300 10.3816/CLM.2006.n.003 16507206

[B38] Harvey RA Champe PC Fisher BD Lippincott’s Illustrated Reviews: Microbiology 2007 2 Philadelphia: Lippincott Williams & Wilkins

[B39] Fredlund H Falk L Jurstrand M Unemo M Molecular genetic methods for diagnosis and characterisation of Chlamydia trachomatis and Neisseria gonorrhoeae: impact on epidemiological surveillance and interventions APMIS 2004 112 771 784 10.1111/j.1600-0463.2004.apm11211-1205.x 15638837

[B40] Berdichevski T Barshack I Bar-Meir S Ben-Horin S Pseudomembranes in a patient with flare-up of inflammatory bowel disease (IBD): is it only clostridium difficile or is it still an IBD exacerbation? Endoscopy 2010 42 Suppl 2 E131 20405379 10.1055/s-0029-1244045

[B41] Satterfield BA Stewart AF Lew CS Pickett DO Cohen MN Moore EA Luedtke PF O’Neill KL Robison RA A quadruplex real-time PCR assay for rapid detection and differentiation of the Clostridium botulinum toxin genes A, B, E and F J Med Microbiol 2010 59 55 64 10.1099/jmm.0.012567-0 19779029

[B42] Brook I The role of anaerobic bacteria in cutaneous and soft tissue abscesses and infected cysts Anaerobe 2007 13 171 177 10.1016/j.anaerobe.2007.08.004 17923425

[B43] DeSantis TZ Hugenholtz P Larsen N Rojas M Brodie EL Keller K Huber T Dalevi D Hu P Andersen GL Greengenes, a chimera-checked 16S rRNA gene database and workbench compatible with ARB Appl Environ Microbiol 2006 72 5069 5072 10.1128/AEM.03006-05 16820507 PMC1489311

[B44] Quast C Pruesse E Yilmaz P Gerken J Schweer T Yarza P Peplies J Glockner FO The SILVA ribosomal RNA gene database project: improved data processing and web-based tools Nucleic Acids Res 2013 41 D590 D596 10.1093/nar/gks1219 23193283 PMC3531112

[B45] Wang WC Lin FM Chang WC Lin KY Huang HD Lin NS miRExpress: analyzing high-throughput sequencing data for profiling microRNA expression BMC Bioinformatics 2009 10 328 10.1186/1471-2105-10-328 19821977 PMC2767369

[B46] Li H Handsaker B Wysoker A Fennell T Ruan J Homer N Marth G Abecasis G Durbin R Genome Project Data Processing S The sequence alignment/map format and SAMtools Bioinformatics 2009 25 2078 2079 10.1093/bioinformatics/btp352 19505943 PMC2723002

[B47] Chmielewska A Szajewska H Systematic review of randomised controlled trials: probiotics for functional constipation World J Gastroenterol 2010 16 69 75 20039451 10.3748/wjg.v16.i1.69 PMC2799919

[B48] Chassard C Dapoigny M Scott KP Crouzet L Del’homme C Marquet P Martin JC Pickering G Ardid D Eschalier A Functional dysbiosis within the gut microbiota of patients with constipated-irritable bowel syndrome Aliment Pharmacol Ther 2012 35 828 838 10.1111/j.1365-2036.2012.05007.x 22315951

[B49] Schwiertz A Taras D Schafer K Beijer S Bos NA Donus C Hardt PD Microbiota and SCFA in lean and overweight healthy subjects Obesity (Silver Spring) 2010 18 190 195 10.1038/oby.2009.167 19498350

[B50] Balamurugan R George G Kabeerdoss J Hepsiba J Chandragunasekaran AM Ramakrishna BS Quantitative differences in intestinal Faecalibacterium prausnitzii in obese Indian children Br J Nutr 2010 103 335 338 10.1017/S0007114509992182 19849869

[B51] Million M Maraninchi M Henry M Armougom F Richet H Carrieri P Valero R Raccah D Vialettes B Raoult D Obesity-associated gut microbiota is enriched in lactobacillus reuteri and depleted in Bifidobacterium animalis and methanobrevibacter smithii Int J Obes (Lond) 2012 36 817 825 10.1038/ijo.2011.153 21829158 PMC3374072

[B52] Armougom F Henry M Vialettes B Raccah D Raoult D Monitoring bacterial community of human gut microbiota reveals an increase in lactobacillus in obese patients and methanogens in anorexic patients PloS one 2009 4 e7125 10.1371/journal.pone.0007125 19774074 PMC2742902

[B53] Noor SO Ridgway K Scovell L Kemsley EK Lund EK Jamieson C Johnson IT Narbad A Ulcerative colitis and irritable bowel patients exhibit distinct abnormalities of the gut microbiota BMC Gastroenterol 2010 10 134 10.1186/1471-230X-10-134 21073731 PMC3002299

[B54] Carroll IM Ringel-Kulka T Siddle JP Ringel Y Alterations in composition and diversity of the intestinal microbiota in patients with diarrhea-predominant irritable bowel syndrome Neurogastroenterol Motil 2012 24 521 530 e248 10.1111/j.1365-2982.2012.01891.x 22339879 PMC3975596

[B55] Duboc H Rainteau D Rajca S Humbert L Farabos D Maubert M Grondin V Jouet P Bouhassira D Seksik P Increase in fecal primary bile acids and dysbiosis in patients with diarrhea-predominant irritable bowel syndrome Neurogastroenterol Motil 2012 24 513 520 e246-517 10.1111/j.1365-2982.2012.01893.x 22356587

[B56] Kerckhoffs AP Ben-Amor K Samsom M van der Rest ME de Vogel J Knol J Akkermans LM Molecular analysis of faecal and duodenal samples reveals significantly higher prevalence and numbers of Pseudomonas aeruginosa in irritable bowel syndrome J Med Microbiol 2011 60 236 245 10.1099/jmm.0.022848-0 20947663

[B57] Saulnier DM Riehle K Mistretta TA Diaz MA Mandal D Raza S Weidler EM Qin X Coarfa C Milosavljevic A Gastrointestinal microbiome signatures of pediatric patients with irritable bowel syndrome Gastroenterology 2011 141 1782 1791 10.1053/j.gastro.2011.06.072 21741921 PMC3417828

[B58] Rajilic-Stojanovic M Biagi E Heilig HG Kajander K Kekkonen RA Tims S de Vos WM Global and deep molecular analysis of microbiota signatures in fecal samples from patients with irritable bowel syndrome Gastroenterology 2011 141 1792 1801 10.1053/j.gastro.2011.07.043 21820992

[B59] Sokol H Seksik P Furet JP Firmesse O Nion-Larmurier I Beaugerie L Cosnes J Corthier G Marteau P Dore J Low counts of faecalibacterium prausnitzii in colitis microbiota Inflamm Bowel Dis 2009 15 1183 1189 10.1002/ibd.20903 19235886

[B60] Andoh A Imaeda H Aomatsu T Inatomi O Bamba S Sasaki M Saito Y Tsujikawa T Fujiyama Y Comparison of the fecal microbiota profiles between ulcerative colitis and Crohn’s disease using terminal restriction fragment length polymorphism analysis J Gastroenterol 2011 46 479 486 10.1007/s00535-010-0368-4 21253779

[B61] Sokol H Lepage P Seksik P Dore J Marteau P Temperature gradient gel electrophoresis of fecal 16S rRNA reveals active Escherichia coli in the microbiota of patients with ulcerative colitis J Clin Microbiol 2006 44 3172 3177 10.1128/JCM.02600-05 16954244 PMC1594675

[B62] Wang T Cai G Qiu Y Fei N Zhang M Pang X Jia W Cai S Zhao L Structural segregation of gut microbiota between colorectal cancer patients and healthy volunteers ISME J 2012 6 320 329 10.1038/ismej.2011.109 21850056 PMC3260502

[B63] Moore WE Moore LH Intestinal floras of populations that have a high risk of colon cancer Appl Environ Microbiol 1995 61 3202 3207 7574628 10.1128/aem.61.9.3202-3207.1995 PMC167598

[B64] Shen XJ Rawls JF Randall T Burcal L Mpande CN Jenkins N Jovov B Abdo Z Sandler RS Keku TO Molecular characterization of mucosal adherent bacteria and associations with colorectal adenomas Gut Microbes 2010 1 138 147 10.4161/gmic.1.3.12360 20740058 PMC2927011

[B65] Mcgill R Tukey JW Larsen WA Variations of box plots Am Stat 1978 32 12 16

