# Supplementary material for: Clinical detection of human probiotics and human pathogenic bacteria by using a novel high-throughput platform based on next generation sequencing
Source: J Clin Bioinforma. 2014 Jan 13;4:1. doi: 10.1186/2043-9113-4-1 (PMC3901789; doi:10.1186/2043-9113-4-1)
Supplement: Additional file 1 — The list of probiotics and pathogens were obtained from literatures or the claims of official departments: Table S1. The reference list of probiotics. Table S2. The reference list of pathogens. [file 2043-9113-4-1-S1.docx]

### Additional file 1

## Table S1 - The reference list of probiotics.

| Probiotics | Reference |
| --- | --- |
| *Bacillus coagulans* | [[1-3](#_ENREF_1)] |
| *Bifidobacterium adolescentis* | [[2-4](#_ENREF_2)] |
| *Bifidobacterium animalis* | [[2-6](#_ENREF_2)] |
| *Bifidobacterium bifidum* | [[2-4](#_ENREF_2), [6](#_ENREF_6), [7](#_ENREF_7)] |
| *Bifidobacterium breve* | [[2-4](#_ENREF_2)] |
| *Bifidobacterium longum* | [[2-4](#_ENREF_2), [6](#_ENREF_6)] |
| *Lactobacillus acidophilus* | [[2-8](#_ENREF_2)] |
| *Lactobacillus amylovorus* | [[4](#_ENREF_4), [9](#_ENREF_9)] |
| *Lactobacillus brevis* | [[3](#_ENREF_3), [4](#_ENREF_4)] |
| *Lactobacillus casei* | [[2-7](#_ENREF_2)] |
| *Lactobacillus crispatus* | [[4](#_ENREF_4), [10](#_ENREF_10)] |
| *Lactobacillus fermentum* | [[2](#_ENREF_2), [3](#_ENREF_3), [6](#_ENREF_6), [9](#_ENREF_9)] |
| *Lactobacillus gasseri* | [[2-4](#_ENREF_2)] |
| *Lactobacillus johnsonii* | [[2-4](#_ENREF_2)] |
| *Lactobacillus paracasei* | [[2-4](#_ENREF_2)] |
| *Lactobacillus plantarum* | [[2-4](#_ENREF_2), [6](#_ENREF_6)] |
| *Lactobacillus reuteri* | [[3-5](#_ENREF_3)] |
| *Lactobacillus rhamnosus* | [[2-7](#_ENREF_2), [11](#_ENREF_11)] |
| *Lactobacillus salivarius* | [[2](#_ENREF_2), [3](#_ENREF_3), [6](#_ENREF_6)] |
| *Lactococcus lactis* | [[3](#_ENREF_3), [7](#_ENREF_7)] |
| *Streptococcus thermophilus* | [[3](#_ENREF_3), [5](#_ENREF_5), [7](#_ENREF_7)] |

## Table S2- The reference list of pathogens.

| Pathogenes | Reference |
| --- | --- |
| *Acinetobacter baumannii*  *Actinomyces bovis*  *Actinomyces israelii*  *Actinomyces naeslundii*  *Actinomyces viscosus*  *Aggregatibacter actinomycetemcomitans*  *Bacillus anthracis*  *Bacillus cereus*  *Bacillus licheniformis*  *Bartonella henselae*  *Bordetella parapertusis* | [[12](#_ENREF_12), [13](#_ENREF_13)] |
| *Actinomyces bovis* | [[14](#_ENREF_14), [15](#_ENREF_15)] |
| *Actinomyces israelii* | [[16](#_ENREF_16), [17](#_ENREF_17)] |
| *Actinomyces naeslundii* | [[18](#_ENREF_18), [19](#_ENREF_19)] |
| *Actinomyces viscosus* | [[18](#_ENREF_18), [19](#_ENREF_19)] |
| *Aggregatibacter actinomycetemcomitans* | [[20](#_ENREF_20), [21](#_ENREF_21)] |
| *Bacillus anthracis* | [[22](#_ENREF_22), [23](#_ENREF_23)] |
| *Bacillus cereus* | [[24](#_ENREF_24), [25](#_ENREF_25)] |
| *Bacillus licheniformis* | [[26](#_ENREF_26), [27](#_ENREF_27)] |
| *Bartonella henselae* | [[28](#_ENREF_28), [29](#_ENREF_29)] |
| *Bordetella parapertussis* | [[30](#_ENREF_30), [31](#_ENREF_31)] |
| *Bordetella pertussis* | [[31-33](#_ENREF_31)] |
| *Borrelia burgdorferi* | [[34](#_ENREF_34), [35](#_ENREF_35)] |
| *Borrelia recurrentis* | [[36](#_ENREF_36), [37](#_ENREF_37)] |
| *Brucella abortus* | [[33](#_ENREF_33), [38](#_ENREF_38), [39](#_ENREF_39)] |
| *Brucella canis* | [[40](#_ENREF_40), [41](#_ENREF_41)] |
| *Brucella melitensis* | [[33](#_ENREF_33), [38](#_ENREF_38), [39](#_ENREF_39)] |
| *Brucella suis* | [[42](#_ENREF_42), [43](#_ENREF_43)] |
| *Burkholderia mallei* | [[44](#_ENREF_44), [45](#_ENREF_45)] |
| *Burkholderia pseudomallei* | [[44](#_ENREF_44), [45](#_ENREF_45)] |
| *Campylobacter coli* | [[46](#_ENREF_46), [47](#_ENREF_47)] |
| *Campylobacter jejuni* | [[3](#_ENREF_3), [33](#_ENREF_33), [46-48](#_ENREF_46)] |
| *Cardiobacterium hominis* | [[49](#_ENREF_49), [50](#_ENREF_50)] |
| *Chlamydia pneumoniae* | [[33](#_ENREF_33), [51](#_ENREF_51)] |
| *Chlamydia psittaci* | [[52](#_ENREF_52), [53](#_ENREF_53)] |
| *Chlamydia trachomatis* | [[33](#_ENREF_33), [53-55](#_ENREF_53)] |
| *Clostridium botulinum* | [[3](#_ENREF_3), [33](#_ENREF_33), [56](#_ENREF_56), [57](#_ENREF_57)] |
| *Clostridium difficile* | [[33](#_ENREF_33), [56](#_ENREF_56), [58](#_ENREF_58)] |
| *Clostridium perfringens* | [[33](#_ENREF_33), [56](#_ENREF_56)] |
| *Clostridium tetani* | [[33](#_ENREF_33), [56](#_ENREF_56)] |
| *Corynebacterium diphtheriae* | [[33](#_ENREF_33), [59](#_ENREF_59)] |
| *Coxiella burnetti* | [[60](#_ENREF_60), [61](#_ENREF_61)] |
| *Ehrlichia canis* | [[62-64](#_ENREF_62)] |
| *Ehrlichia chaffeensis* | [[62](#_ENREF_62), [64](#_ENREF_64), [65](#_ENREF_65)] |
| *Ehrlichia ewingii* | [[62](#_ENREF_62), [64](#_ENREF_64), [66](#_ENREF_66)] |
| *Eikenella corrodens* | [[67](#_ENREF_67), [68](#_ENREF_68)] |
| *Enterococcus faecalis* | [[33](#_ENREF_33), [69](#_ENREF_69)] |
| *Enterococcus faecium* | [[33](#_ENREF_33), [70](#_ENREF_70)] |
| *Erysipelothrix rhusiopathiae* | [[71](#_ENREF_71), [72](#_ENREF_72)] |
| *Escherichia coli* | [[33](#_ENREF_33), [56](#_ENREF_56)] |
| *Francisella tularensis* | [[73](#_ENREF_73), [74](#_ENREF_74)] |
| *Gardnerella vaginalis* | [[75-78](#_ENREF_75)] |
| *Haemophilus ducreyi* | [[79-81](#_ENREF_79)] |
| *Haemophilus influenzae* | [[33](#_ENREF_33), [82](#_ENREF_82), [83](#_ENREF_83)] |
| *Haemophilus parainfluenzae* | [[83](#_ENREF_83), [84](#_ENREF_84)] |
| *Helicobacter pylori* | [[33](#_ENREF_33), [85](#_ENREF_85)] |
| *Kingella kingae* | [[86](#_ENREF_86), [87](#_ENREF_87)] |
| *Klebsiella granulomatis* | [[81](#_ENREF_81), [88](#_ENREF_88)] |
| *Klebsiella pneumoniae* | [[89](#_ENREF_89), [90](#_ENREF_90)] |
| *Legionella pneumophila* | [[33](#_ENREF_33), [91](#_ENREF_91)] |
| *Leptospira interrogans* | [[92](#_ENREF_92), [93](#_ENREF_93)] |
| *Leptospira santarosai* | [[94](#_ENREF_94), [95](#_ENREF_95)] |
| *Listeria ivanovii* | [[96](#_ENREF_96), [97](#_ENREF_97)] |
| *Listeria monocytogenes* | [[33](#_ENREF_33), [96](#_ENREF_96), [98](#_ENREF_98), [99](#_ENREF_99)] |
| *Mycobacterium bovis* | [[100-103](#_ENREF_100)] |
| *Mycobacterium caprae* | [[102-104](#_ENREF_102)] |
| *Mycobacterium leprae* | [[105](#_ENREF_105), [106](#_ENREF_106)] |
| *Mycobacterium pinnipedii* | [[103](#_ENREF_103), [107](#_ENREF_107)] |
| *Mycobacterium tuberculosis* | [[103](#_ENREF_103), [108](#_ENREF_108), [109](#_ENREF_109)] |
| *Mycoplasma genitalium* | [[110](#_ENREF_110), [111](#_ENREF_111)] |
| *Mycoplasma hominis* | [[78](#_ENREF_78), [112](#_ENREF_112)] |
| *Neisseria gonorrhoeae* | [[33](#_ENREF_33), [54](#_ENREF_54), [55](#_ENREF_55), [113](#_ENREF_113)] |
| *Neisseria meningitidis* | [[33](#_ENREF_33), [113-115](#_ENREF_113)] |
| *Nocardia asteroides* | [[116](#_ENREF_116), [117](#_ENREF_117)] |
| *Nocardia brasiliensis* | [[117](#_ENREF_117), [118](#_ENREF_118)] |
| *Orientia tsutsugamushi* | [[119](#_ENREF_119), [120](#_ENREF_120)] |
| *Pasteurella multocida* | [[121](#_ENREF_121), [122](#_ENREF_122)] |
| *Pseudomonas aeruginosa* | [[33](#_ENREF_33), [56](#_ENREF_56), [123](#_ENREF_123), [124](#_ENREF_124)] |
| *Rickettsia akari* | [[125](#_ENREF_125), [126](#_ENREF_126)] |
| *Rickettsia australis* | [[127](#_ENREF_127), [128](#_ENREF_128)] |
| *Rickettsia conorii* | [[99](#_ENREF_99), [129](#_ENREF_129)] |
| *Rickettsia prowazeki* | [[130](#_ENREF_130), [131](#_ENREF_131)] |
| *Rickettsia rickettsii* | [[132](#_ENREF_132), [133](#_ENREF_133)] |
| *Rickettsia sibirica* | [[134-136](#_ENREF_134)] |
| *Rickettsia typhi* | [[137](#_ENREF_137), [138](#_ENREF_138)] |
| *Rothia mucilaginosa* | [[139](#_ENREF_139), [140](#_ENREF_140)] |
| *Salmonella enterica* | [[3](#_ENREF_3), [33](#_ENREF_33), [141-143](#_ENREF_141)] |
| *Shigella boydii* | [[144](#_ENREF_144), [145](#_ENREF_145)] |
| *Shigella dysenteriae* | [[146](#_ENREF_146), [147](#_ENREF_147)] |
| *Shigella flexneri* | [[99](#_ENREF_99), [148](#_ENREF_148), [149](#_ENREF_149)] |
| *Shigella sonnei* | [[33](#_ENREF_33), [150](#_ENREF_150)] |
| *Spirillum minus* | [[151](#_ENREF_151), [152](#_ENREF_152)] |
| *Staphylococcus aureus* | [[3](#_ENREF_3), [33](#_ENREF_33), [56](#_ENREF_56), [153](#_ENREF_153), [154](#_ENREF_154)] |
| *Staphylococcus epidermidis* | [[33](#_ENREF_33), [155](#_ENREF_155), [156](#_ENREF_156)] |
| *Staphylococcus lugdunensis* | [[157](#_ENREF_157), [158](#_ENREF_158)] |
| *Staphylococcus saprophyticus* | [[159](#_ENREF_159), [160](#_ENREF_160)] |
| *Streptobacillus moniliformis* | [[161](#_ENREF_161), [162](#_ENREF_162)] |
| *Streptococcus agalactiae* | [[33](#_ENREF_33), [56](#_ENREF_56), [163](#_ENREF_163), [164](#_ENREF_164)] |
| *Streptococcus pneumoniae* | [[33](#_ENREF_33), [56](#_ENREF_56), [165](#_ENREF_165), [166](#_ENREF_166)] |
| *Streptococcus pyogenes* | [[33](#_ENREF_33), [56](#_ENREF_56), [167](#_ENREF_167), [168](#_ENREF_168)] |
| *Streptococcus suis* | [[169](#_ENREF_169), [170](#_ENREF_170)] |
| *Treponema pallidum* | [[81](#_ENREF_81), [171](#_ENREF_171), [172](#_ENREF_172)] |
| *Ureaplasma urealyticum* | [[112](#_ENREF_112), [173](#_ENREF_173), [174](#_ENREF_174)] |
| *Vibrio cholerae* | [[3](#_ENREF_3), [33](#_ENREF_33), [175-177](#_ENREF_175)] |
| *Vibrio parahaemolyticus* | [[178](#_ENREF_178), [179](#_ENREF_179)] |
| *Vibrio vulnificus* | [[180](#_ENREF_180), [181](#_ENREF_181)] |
| *Yersinia enterocolitica* | [[182](#_ENREF_182), [183](#_ENREF_183)] |
| *Yersinia pestis* | [[33](#_ENREF_33), [184](#_ENREF_184)] |
| *Yersinia pseudotuberculosis* | [[185](#_ENREF_185), [186](#_ENREF_186)] |

### References

1. Hun L: **Bacillus coagulans significantly improved abdominal pain and bloating in patients with IBS.** *Postgraduate medicine* 2009, **121:**119-124.

2. **Health Canada** [<http://www.hc-sc.gc.ca/fn-an/label-etiquet/claims-reclam/probiotics_claims-allegations_probiotiques-eng.php>]

3. **Taiwan Food and Drug Administration** [<http://consumer.fda.gov.tw/Food/Material.aspx?nodeID=160>]

4. Gilliland SE: **Technological & Commercial Applications of Lactic Acid Bacteria; Health & Nutritional Benefits in Dairy Products.** In *Book Technological & Commercial Applications of Lactic Acid Bacteria; Health & Nutritional Benefits in Dairy Products* (Editor ed.^eds.). City.

5. Saulnier DM, Spinler JK, Gibson GR, Versalovic J: **Mechanisms of probiosis and prebiosis: considerations for enhanced functional foods.** *Current opinion in biotechnology* 2009, **20:**135-141.

6. Gill H, Prasad J: **Probiotics, immunomodulation, and health benefits.** *Advances in experimental medicine and biology* 2008, **606:**423-454.

7. Salminen S, von Wright A, Morelli L, Marteau P, Brassart D, de Vos WM, Fonden R, Saxelin M, Collins K, Mogensen G, et al: **Demonstration of safety of probiotics -- a review.** *International journal of food microbiology* 1998, **44:**93-106.

8. Lenoir-Wijnkoop I, Sanders ME, Cabana MD, Caglar E, Corthier G, Rayes N, Sherman PM, Timmerman HM, Vaneechoutte M, Van Loo J, Wolvers DA: **Probiotic and prebiotic influence beyond the intestinal tract.** *Nutrition reviews* 2007, **65:**469-489.

9. Omar JM, Chan YM, Jones ML, Prakash S, Jones PJH: **Lactobacillus fermentum and Lactobacillus amylovorus as probiotics alter body adiposity and gut microflora in healthy persons.** *J Funct Foods* 2013, **5:**116-123.

10. Castagliuolo I, Galeazzi F, Ferrari S, Elli M, Brun P, Cavaggioni A, Tormen D, Sturniolo GC, Morelli L, Palu G: **Beneficial effect of auto-aggregating Lactobacillus crispatus on experimentally induced colitis in mice.** *Fems Immunol Med Mic* 2005, **43:**197-204.

11. Reid G, Jass J, Sebulsky MT, McCormick JK: **Potential uses of probiotics in clinical practice.** *Clinical microbiology reviews* 2003, **16:**658-672.

12. Dijkshoorn L, Nemec A, Seifert H: **An increasing threat in hospitals: multidrug-resistant Acinetobacter baumannii.** *Nat Rev Microbiol* 2007, **5:**939-951.

13. Gouby A, Carles-Nurit MJ, Bouziges N, Bourg G, Mesnard R, Bouvet PJ: **Use of pulsed-field gel electrophoresis for investigation of hospital outbreaks of Acinetobacter baumannii.** *Journal of Clinical Microbiology* 1992, **30:**1588-1591.

14. Mansouri P, Farshi S, Khosravi A, Naraghi ZS: **Primary cutaneous actinomycosis caused by Actinomyces bovis in a patient with common variable immunodeficiency.** *J Dermatol* 2011, **38:**911-915.

15. Sudhakar SS, Ross JJ: **Short-term treatment of actinomycosis: two cases and a review.** *Clin Infect Dis* 2004, **38:**444-447.

16. Spence MR, Gupta PK, Frost JK, King TM: **Cytologic Detection and Clinical Significance of Actinomyces-Israelii in Women Using Intra-Uterine Contraceptive Devices.** *Am J Obstet Gynecol* 1978, **131:**295-298.

17. Persson E, Holmberg K: **A longitudinal study of Actinomyces israelii in the female genital tract.** *Acta Obstet Gynecol Scand* 1984, **63:**207-216.

18. Costello AH, Cisar JO, Kolenbrander PE, Gabriel O: **Neuraminidase-dependent hamagglutination of human erythrocytes by human strains of Actinomyces viscosus and Actinomyces naeslundii.** *Infect Immun* 1979, **26:**563-572.

19. Cisar JO, Kolenbrander PE, McIntire FC: **Specificity of coaggregation reactions between human oral streptococci and strains of Actinomyces viscosus or Actinomyces naeslundii.** *Infect Immun* 1979, **24:**742-752.

20. Kachlany SC: **Aggregatibacter actinomycetemcomitans leukotoxin: from threat to therapy.** *J Dent Res* 2010, **89:**561-570.

21. Fine DH, Markowitz K, Furgang D, Fairlie K, Ferrandiz J, Nasri C, McKiernan M, Gunsolley J: **Aggregatibacter actinomycetemcomitans and its relationship to initiation of localized aggressive periodontitis: longitudinal cohort study of initially healthy adolescents.** *Journal of Clinical Microbiology* 2007, **45:**3859-3869.

22. Schuch R, Nelson D, Fischetti VA: **A bacteriolytic agent that detects and kills Bacillus anthracis.** *Nature* 2002, **418:**884-889.

23. Mikesell P, Ivins BE, Ristroph JD, Dreier TM: **Evidence for plasmid-mediated toxin production in Bacillus anthracis.** *Infect Immun* 1983, **39:**371-376.

24. Lapidus A, Goltsman E, Auger S, Galleron N, Segurens B, Dossat C, Land ML, Broussolle V, Brillard J, Guinebretiere MH, et al: **Extending the Bacillus cereus group genomics to putative food-borne pathogens of different toxicity.** *Chem Biol Interact* 2008, **171:**236-249.

25. Granum PE, Lund T: **Bacillus cereus and its food poisoning toxins.** *FEMS Microbiol Lett* 1997, **157:**223-228.

26. Salkinoja-Salonen MS, Vuorio R, Andersson MA, Kampfer P, Andersson MC, Honkanen-Buzalski T, Scoging AC: **Toxigenic strains of Bacillus licheniformis related to food poisoning.** *Appl Environ Microbiol* 1999, **65:**4637-4645.

27. Neugebauer K, Sprengel R, Schaller H: **Penicillinase from Bacillus licheniformis: nucleotide sequence of the gene and implications for the biosynthesis of a secretory protein in a Gram-positive bacterium.** *Nucleic Acids Res* 1981, **9:**2577-2588.

28. Chomel BB, Kasten RW, Floyd-Hawkins K, Chi B, Yamamoto K, Roberts-Wilson J, Gurfield AN, Abbott RC, Pedersen NC, Koehler JE: **Experimental transmission of Bartonella henselae by the cat flea.** *Journal of Clinical Microbiology* 1996, **34:**1952-1956.

29. Drancourt M, Birtles R, Chaumentin G, Vandenesch F, Etienne J, Raoult D: **New serotype of Bartonella henselae in endocarditis and cat-scratch disease.** *Lancet* 1996, **347:**441-443.

30. Heininger U, Stehr K, Schmitt-Grohe S, Lorenz C, Rost R, Christenson PD, Uberall M, Cherry JD: **Clinical characteristics of illness caused by Bordetella parapertussis compared with illness caused by Bordetella pertussis.** *Pediatr Infect Dis J* 1994, **13:**306-309.

31. He Q, Viljanen MK, Arvilommi H, Aittanen B, Mertsola J: **Whooping cough caused by Bordetella pertussis and Bordetella parapertussis in an immunized population.** *JAMA* 1998, **280:**635-637.

32. Kenneth Todar PD: **Bordetella pertussis and Whooping Cough.** In *Book Bordetella pertussis and Whooping Cough* (Editor ed.^eds.). City; 2008.

33. Richard A. Harvey Ph.D. PCC, Bruce D. Fisher: *Lippincott's Illustrated Reviews: Microbiology.* Second edn; 2007.

34. Casjens S, Palmer N, van Vugt R, Huang WM, Stevenson B, Rosa P, Lathigra R, Sutton G, Peterson J, Dodson RJ, et al: **A bacterial genome in flux: the twelve linear and nine circular extrachromosomal DNAs in an infectious isolate of the Lyme disease spirochete Borrelia burgdorferi.** *Mol Microbiol* 2000, **35:**490-516.

35. Fraser CM, Casjens S, Huang WM, Sutton GG, Clayton R, Lathigra R, White O, Ketchum KA, Dodson R, Hickey EK, et al: **Genomic sequence of a Lyme disease spirochaete, Borrelia burgdorferi.** *Nature* 1997, **390:**580-586.

36. Lescot M, Audic S, Robert C, Nguyen TT, Blanc G, Cutler SJ, Wincker P, Couloux A, Claverie JM, Raoult D, Drancourt M: **The genome of Borrelia recurrentis, the agent of deadly louse-borne relapsing fever, is a degraded subset of tick-borne Borrelia duttonii.** *PLoS Genet* 2008, **4:**e1000185.

37. Butler T, Hazen P, Wallace CK, Awoke S, Habte-Michael A: **Infection with Borrelia recurrentis: pathogenesis of fever and petechiae.** *J Infect Dis* 1979, **140:**665-675.

38. Maley MW, Kociuba K, Chan RC: **Prevention of laboratory-acquired brucellosis: significant side effects of prophylaxis.** *Clin Infect Dis* 2006, **42:**433-434.

39. Seleem MN, Boyle SM, Sriranganathan N: **Brucella: a pathogen without classic virulence genes.** *Veterinary microbiology* 2008, **129:**1-14.

40. Lucero NE, Escobar GI, Ayala SM, Jacob N: **Diagnosis of human brucellosis caused by Brucella canis.** *Journal of Medical Microbiology* 2005, **54:**457-461.

41. Polt SS, Dismukes WE, Flint A, Schaefer J: **Human brucellosis caused by Brucella canis: clinical features and immune response.** *Ann Intern Med* 1982, **97:**717-719.

42. Kohler S, Foulongne V, Ouahrani-Bettache S, Bourg G, Teyssier J, Ramuz M, Liautard JP: **The analysis of the intramacrophagic virulome of Brucella suis deciphers the environment encountered by the pathogen inside the macrophage host cell.** *Proc Natl Acad Sci U S A* 2002, **99:**15711-15716.

43. Gross A, Terraza A, Ouahrani-Bettache S, Liautard JP, Dornand J: **In vitro Brucella suis infection prevents the programmed cell death of human monocytic cells.** *Infect Immun* 2000, **68:**342-351.

44. Galyov EE, Brett PJ, DeShazer D: **Molecular insights into Burkholderia pseudomallei and Burkholderia mallei pathogenesis.** *Annu Rev Microbiol* 2010, **64:**495-517.

45. Godoy D, Randle G, Simpson AJ, Aanensen DM, Pitt TL, Kinoshita R, Spratt BG: **Multilocus sequence typing and evolutionary relationships among the causative agents of melioidosis and glanders, Burkholderia pseudomallei and Burkholderia mallei.** *Journal of Clinical Microbiology* 2003, **41:**2068-2079.

46. Linton D, Lawson AJ, Owen RJ, Stanley J: **PCR detection, identification to species level, and fingerprinting of Campylobacter jejuni and Campylobacter coli direct from diarrheic samples.** *Journal of Clinical Microbiology* 1997, **35:**2568-2572.

47. Gonzalez I, Grant KA, Richardson PT, Park SF, Collins MD: **Specific identification of the enteropathogens Campylobacter jejuni and Campylobacter coli by using a PCR test based on the ceuE gene encoding a putative virulence determinant.** *Journal of Clinical Microbiology* 1997, **35:**759-763.

48. Parkhill J, Wren BW, Mungall K, Ketley JM, Churcher C, Basham D, Chillingworth T, Davies RM, Feltwell T, Holroyd S, et al: **The genome sequence of the food-borne pathogen Campylobacter jejuni reveals hypervariable sequences.** *Nature* 2000, **403:**665-668.

49. Malani AN, Aronoff DM, Bradley SF, Kauffman CA: **Cardiobacterium hominis endocarditis: Two cases and a review of the literature.** *Eur J Clin Microbiol Infect Dis* 2006, **25:**587-595.

50. Francioli PB, Roussianos D, Glauser MP: **Cardiobacterium hominis endocarditis manifesting as bacterial meningitis.** *Arch Intern Med* 1983, **143:**1483-1484.

51. Zhan P, Suo LJ, Qian Q, Shen XK, Qiu LX, Yu LK, Song Y: **Chlamydia pneumoniae infection and lung cancer risk: a meta-analysis.** *Eur J Cancer* 2011, **47:**742-747.

52. Ferreri AJ, Guidoboni M, Ponzoni M, De Conciliis C, Dell'Oro S, Fleischhauer K, Caggiari L, Lettini AA, Dal Cin E, Ieri R, et al: **Evidence for an association between Chlamydia psittaci and ocular adnexal lymphomas.** *J Natl Cancer Inst* 2004, **96:**586-594.

53. Byrne GI, Moulder JW: **Parasite-specified phagocytosis of Chlamydia psittaci and Chlamydia trachomatis by L and HeLa cells.** *Infect Immun* 1978, **19:**598-606.

54. Fredlund H, Falk L, Jurstrand M, Unemo M: **Molecular genetic methods for diagnosis and characterisation of Chlamydia trachomatis and Neisseria gonorrhoeae: impact on epidemiological surveillance and interventions.** *APMIS : acta pathologica, microbiologica, et immunologica Scandinavica* 2004, **112:**771-784.

55. Cook RL, Hutchison SL, Ostergaard L, Braithwaite RS, Ness RB: **Systematic review: noninvasive testing for Chlamydia trachomatis and Neisseria gonorrhoeae.** *Ann Intern Med* 2005, **142:**914-925.

56. Brook I: **The role of anaerobic bacteria in cutaneous and soft tissue abscesses and infected cysts.** *Anaerobe* 2007, **13:**171-177.

57. Satterfield BA, Stewart AF, Lew CS, Pickett DO, Cohen MN, Moore EA, Luedtke PF, O'Neill KL, Robison RA: **A quadruplex real-time PCR assay for rapid detection and differentiation of the Clostridium botulinum toxin genes A, B, E and F.** *Journal of Medical Microbiology* 2010, **59:**55-64.

58. Berdichevski T, Barshack I, Bar-Meir S, Ben-Horin S: **Pseudomembranes in a patient with flare-up of inflammatory bowel disease (IBD): is it only Clostridium difficile or is it still an IBD exacerbation?** *Endoscopy* 2010, **42 Suppl 2:**E131.

59. Nester EW, Anderson DG, Roberts JCE, Pearsall NN, Nester MT: *Microbiology: A Human Perspective.* Fourth edn. Boston: McGraw-Hill; 2003.

60. Fenollar F, Fournier PE, Raoult D: **Molecular detection of Coxiella burnetii in the sera of patients with Q fever endocarditis or vascular infection.** *Journal of Clinical Microbiology* 2004, **42:**4919-4924.

61. Urbano-Marquez A, Grau Junyent JM, Valls Arara V, Periz Sague A, Cardellach F, Navarro Lopez F, Revert L, Rozman C: **[Endocarditis by Coxiella burnetti. A chronic form of Q fever. Report of one case (author's transl)].** *Med Clin (Barc)* 1979, **73:**242-246.

62. Breitschwerdt EB, Hegarty BC, Hancock SI: **Sequential evaluation of dogs naturally infected with Ehrlichia canis, Ehrlichia chaffeensis, Ehrlichia equi, Ehrlichia ewingii, or Bartonella vinsonii.** *Journal of Clinical Microbiology* 1998, **36:**2645-2651.

63. Maeda K, Markowitz N, Hawley RC, Ristic M, Cox D, McDade JE: **Human infection with Ehrlichia canis, a leukocytic rickettsia.** *N Engl J Med* 1987, **316:**853-856.

64. Rikihisa Y, Ewing SA, Fox JC: **Western immunoblot analysis of Ehrlichia chaffeensis, E. canis, or E. ewingii infections in dogs and humans.** *Journal of Clinical Microbiology* 1994, **32:**2107-2112.

65. Paddock CD, Childs JE: **Ehrlichia chaffeensis: a prototypical emerging pathogen.** *Clinical microbiology reviews* 2003, **16:**37-64.

66. Buller RS, Arens M, Hmiel SP, Paddock CD, Sumner JW, Rikhisa Y, Unver A, Gaudreault-Keener M, Manian FA, Liddell AM, et al: **Ehrlichia ewingii, a newly recognized agent of human ehrlichiosis.** *N Engl J Med* 1999, **341:**148-155.

67. Yumoto H, Nakae H, Fujinaka K, Ebisu S, Matsuo T: **Interleukin-6 (IL-6) and IL-8 are induced in human oral epithelial cells in response to exposure to periodontopathic Eikenella corrodens.** *Infect Immun* 1999, **67:**384-394.

68. Stoloff AL, Gillies ML: **Infections with Eikenella corrodens in a general hospital: a report of 33 cases.** *Rev Infect Dis* 1986, **8:**50-53.

69. Rocas IN, Siqueira JF, Jr., Santos KR: **Association of Enterococcus faecalis with different forms of periradicular diseases.** *J Endod* 2004, **30:**315-320.

70. Leclercq R, Derlot E, Duval J, Courvalin P: **Plasmid-mediated resistance to vancomycin and teicoplanin in Enterococcus faecium.** *N Engl J Med* 1988, **319:**157-161.

71. Brooke CJ, Riley TV: **Erysipelothrix rhusiopathiae: bacteriology, epidemiology and clinical manifestations of an occupational pathogen.** *Journal of Medical Microbiology* 1999, **48:**789-799.

72. Gorby GL, Peacock JE, Jr.: **Erysipelothrix rhusiopathiae endocarditis: microbiologic, epidemiologic, and clinical features of an occupational disease.** *Rev Infect Dis* 1988, **10:**317-325.

73. Larsson P, Elfsmark D, Svensson K, Wikstrom P, Forsman M, Brettin T, Keim P, Johansson A: **Molecular evolutionary consequences of niche restriction in Francisella tularensis, a facultative intracellular pathogen.** *Plos Pathogens* 2009, **5:**e1000472.

74. Larsson P, Oyston PC, Chain P, Chu MC, Duffield M, Fuxelius HH, Garcia E, Halltorp G, Johansson D, Isherwood KE, et al: **The complete genome sequence of Francisella tularensis, the causative agent of tularemia.** *Nat Genet* 2005, **37:**153-159.

75. Taylor E, Blackwell AL, Barlow D, Phillips I: **Gardnerella vaginalis, anaerobes, and vaginal discharge.** *Lancet* 1982, **1:**1376-1379.

76. McDonald HM, O'Loughlin JA, Vigneswaran R, Jolley PT, Harvey JA, Bof A, McDonald PJ: **Impact of metronidazole therapy on preterm birth in women with bacterial vaginosis flora (Gardnerella vaginalis): a randomised, placebo controlled trial.** *Br J Obstet Gynaecol* 1997, **104:**1391-1397.

77. Menard JP, Fenollar F, Henry M, Bretelle F, Raoult D: **Molecular quantification of Gardnerella vaginalis and Atopobium vaginae loads to predict bacterial vaginosis.** *Clin Infect Dis* 2008, **47:**33-43.

78. Zariffard MR, Saifuddin M, Sha BE, Spear GT: **Detection of bacterial vaginosis-related organisms by real-time PCR for Lactobacilli, Gardnerella vaginalis and Mycoplasma hominis.** *Fems Immunol Med Mic* 2002, **34:**277-281.

79. Spinola SM, Wild LM, Apicella MA, Gaspari AA, Campagnari AA: **Experimental human infection with Haemophilus ducreyi.** *J Infect Dis* 1994, **169:**1146-1150.

80. Cope LD, Lumbley S, Latimer JL, Klesney-Tait J, Stevens MK, Johnson LS, Purven M, Munson RS, Jr., Lagergard T, Radolf JD, Hansen EJ: **A diffusible cytotoxin of Haemophilus ducreyi.** *Proc Natl Acad Sci U S A* 1997, **94:**4056-4061.

81. Mackay IM, Harnett G, Jeoffreys N, Bastian I, Sriprakash KS, Siebert D, Sloots TP: **Detection and discrimination of herpes simplex viruses, Haemophilus ducreyi, Treponema pallidum, and Calymmatobacterium (Klebsiella) granulomatis from genital ulcers.** *Clin Infect Dis* 2006, **42:**1431-1438.

82. Eskola J, Peltola H, Takala AK, Kayhty H, Hakulinen M, Karanko V, Kela E, Rekola P, Ronnberg PR, Samuelson JS, et al.: **Efficacy of Haemophilus influenzae type b polysaccharide-diphtheria toxoid conjugate vaccine in infancy.** *N Engl J Med* 1987, **317:**717-722.

83. Sturm AW: **Haemophilus influenzae and Haemophilus parainfluenzae in nongonococcal urethritis.** *J Infect Dis* 1986, **153:**165-167.

84. Suzuki S, Nakatomi Y, Sato H, Tsukada H, Arakawa M: **Haemophilus parainfluenzae antigen and antibody in renal biopsy samples and serum of patients with IgA nephropathy.** *Lancet* 1994, **343:**12-16.

85. Suerbaum S, Michetti P: **Helicobacter pylori infection.** *N Engl J Med* 2002, **347:**1175-1186.

86. Yagupsky P: **Kingella kingae: from medical rarity to an emerging paediatric pathogen.** *Lancet Infect Dis* 2004, **4:**358-367.

87. Goutzmanis JJ, Gonis G, Gilbert GL: **Kingella kingae infection in children: ten cases and a review of the literature.** *Pediatr Infect Dis J* 1991, **10:**677-683.

88. O'Farrell N: **Donovanosis.** *Sex Transm Infect* 2002, **78:**452-457.

89. Nordmann P, Cuzon G, Naas T: **The real threat of Klebsiella pneumoniae carbapenemase-producing bacteria.** *Lancet Infect Dis* 2009, **9:**228-236.

90. Knothe H, Shah P, Krcmery V, Antal M, Mitsuhashi S: **Transferable resistance to cefotaxime, cefoxitin, cefamandole and cefuroxime in clinical isolates of Klebsiella pneumoniae and Serratia marcescens.** *Infection* 1983, **11:**315-317.

91. Rowbotham TJ: **Preliminary report on the pathogenicity of Legionella pneumophila for freshwater and soil amoebae.** *J Clin Pathol* 1980, **33:**1179-1183.

92. Malmstrom J, Beck M, Schmidt A, Lange V, Deutsch EW, Aebersold R: **Proteome-wide cellular protein concentrations of the human pathogen Leptospira interrogans.** *Nature* 2009, **460:**762-765.

93. Ren SX, Fu G, Jiang XG, Zeng R, Miao YG, Xu H, Zhang YX, Xiong H, Lu G, Lu LF, et al: **Unique physiological and pathogenic features of Leptospira interrogans revealed by whole-genome sequencing.** *Nature* 2003, **422:**888-893.

94. Yasuda PH, Steigerwalt AG, Sulzer KR, Kaufmann AF, Rogers F, Brenner DJ: **Deoxyribonucleic-Acid Relatedness between Serogroups and Serovars in the Family Leptospiraceae with Proposals for 7 New Leptospira Species.** *Int J Syst Bacteriol* 1987, **37:**407-415.

95. Hsieh WJ, Chang YF, Chen CS, Pan MJ: **Omp52 is a growth-phase-regulated outer membrane protein of Leptospira santarosai serovar Shermani.** *FEMS Microbiol Lett* 2005, **243:**339-345.

96. Vazquez-Boland JA, Kuhn M, Berche P, Chakraborty T, Dominguez-Bernal G, Goebel W, Gonzalez-Zorn B, Wehland J, Kreft J: **Listeria pathogenesis and molecular virulence determinants.** *Clinical microbiology reviews* 2001, **14:**584-640.

97. Lessing MP, Curtis GD, Bowler IC: **Listeria ivanovii infection.** *J Infect* 1994, **29:**230-231.

98. Farber JM, Peterkin PI: **Listeria monocytogenes, a food-borne pathogen.** *Microbiol Rev* 1991, **55:**476-511.

99. Gouin E, Gantelet H, Egile C, Lasa I, Ohayon H, Villiers V, Gounon P, Sansonetti PJ, Cossart P: **A comparative study of the actin-based motilities of the pathogenic bacteria Listeria monocytogenes, Shigella flexneri and Rickettsia conorii.** *J Cell Sci* 1999, **112 ( Pt 11):**1697-1708.

100. Cosivi O, Grange JM, Daborn CJ, Raviglione MC, Fujikura T, Cousins D, Robinson RA, Huchzermeyer HF, de Kantor I, Meslin FX: **Zoonotic tuberculosis due to Mycobacterium bovis in developing countries.** *Emerg Infect Dis* 1998, **4:**59-70.

101. O'Reilly LM, Daborn CJ: **The epidemiology of Mycobacterium bovis infections in animals and man: a review.** *Tuber Lung Dis* 1995, **76 Suppl 1:**1-46.

102. Rodriguez E, Sanchez LP, Perez S, Herrera L, Jimenez MS, Samper S, Iglesias MJ: **Human tuberculosis due to Mycobacterium bovis and M. caprae in Spain, 2004-2007.** *Int J Tuberc Lung Dis* 2009, **13:**1536-1541.

103. Warren RM, Gey van Pittius NC, Barnard M, Hesseling A, Engelke E, de Kock M, Gutierrez MC, Chege GK, Victor TC, Hoal EG, van Helden PD: **Differentiation of Mycobacterium tuberculosis complex by PCR amplification of genomic regions of difference.** *Int J Tuberc Lung Dis* 2006, **10:**818-822.

104. Prodinger WM, Brandstatter A, Naumann L, Pacciarini M, Kubica T, Boschiroli ML, Aranaz A, Nagy G, Cvetnic Z, Ocepek M, et al: **Characterization of Mycobacterium caprae isolates from Europe by mycobacterial interspersed repetitive unit genotyping.** *Journal of Clinical Microbiology* 2005, **43:**4984-4992.

105. Monot M, Honore N, Garnier T, Zidane N, Sherafi D, Paniz-Mondolfi A, Matsuoka M, Taylor GM, Donoghue HD, Bouwman A, et al: **Comparative genomic and phylogeographic analysis of Mycobacterium leprae.** *Nat Genet* 2009, **41:**1282-1289.

106. Young RA, Mehra V, Sweetser D, Buchanan T, Clark-Curtiss J, Davis RW, Bloom BR: **Genes for the major protein antigens of the leprosy parasite Mycobacterium leprae.** *Nature* 1985, **316:**450-452.

107. Kiers A, Klarenbeek A, Mendelts B, Van Soolingen D, Koeter G: **Transmission of Mycobacterium pinnipedii to humans in a zoo with marine mammals.** *Int J Tuberc Lung Dis* 2008, **12:**1469-1473.

108. Gagneux S, DeRiemer K, Van T, Kato-Maeda M, de Jong BC, Narayanan S, Nicol M, Niemann S, Kremer K, Gutierrez MC, et al: **Variable host-pathogen compatibility in Mycobacterium tuberculosis.** *Proc Natl Acad Sci U S A* 2006, **103:**2869-2873.

109. Cole ST, Brosch R, Parkhill J, Garnier T, Churcher C, Harris D, Gordon SV, Eiglmeier K, Gas S, Barry CE, 3rd, et al: **Deciphering the biology of Mycobacterium tuberculosis from the complete genome sequence.** *Nature* 1998, **393:**537-544.

110. Horner PJ, Gilroy CB, Thomas BJ, Naidoo ROM, Taylorrobinson D: **Association of Mycoplasma-Genitalium with Acute Nongonococcal Urethritis.** *Lancet* 1993, **342:**582-585.

111. Tully JG, Taylorrobinson D, Rose DL, Cole RM, Bove JM: **Mycoplasma-Genitalium, a New Species from the Human Urogenital Tract.** *Int J Syst Bacteriol* 1983, **33:**387-396.

112. Brunner H, Weidner W, Schiefer HG: **Studies on the role of Ureaplasma urealyticum and Mycoplasma hominis in prostatitis.** *J Infect Dis* 1983, **147:**807-813.

113. Petersen BH, Lee TJ, Snyderman R, Brooks GF: **Neisseria meningitidis and Neisseria gonorrhoeae bacteremia associated with C6, C7, or C8 deficiency.** *Ann Intern Med* 1979, **90:**917-920.

114. van Deuren M, Brandtzaeg P, van der Meer JW: **Update on meningococcal disease with emphasis on pathogenesis and clinical management.** *Clinical microbiology reviews* 2000, **13:**144-166, table of contents.

115. Stephens DS, Greenwood B, Brandtzaeg P: **Epidemic meningitis, meningococcaemia, and Neisseria meningitidis.** *Lancet* 2007, **369:**2196-2210.

116. Wallace RJ, Jr., Steele LC, Sumter G, Smith JM: **Antimicrobial susceptibility patterns of Nocardia asteroides.** *Antimicrob Agents Chemother* 1988, **32:**1776-1779.

117. Brown-Elliott BA, Brown JM, Conville PS, Wallace RJ, Jr.: **Clinical and laboratory features of the Nocardia spp. based on current molecular taxonomy.** *Clinical microbiology reviews* 2006, **19:**259-282.

118. Wallace RJ, Jr., Brown BA, Blacklock Z, Ulrich R, Jost K, Brown JM, McNeil MM, Onyi G, Steingrube VA, Gibson J: **New Nocardia taxon among isolates of Nocardia brasiliensis associated with invasive disease.** *Journal of Clinical Microbiology* 1995, **33:**1528-1533.

119. Seong SY, Choi MS, Kim IS: **Orientia tsutsugamushi infection: overview and immune responses.** *Microbes Infect* 2001, **3:**11-21.

120. Nacy CA, Meltzer MS: **Macrophages in resistance to rickettsial infection: macrophage activation in vitro for killing of Rickettsia tsutsugamushi.** *J Immunol* 1979, **123:**2544-2549.

121. Harper M, Boyce JD, Adler B: **Pasteurella multocida pathogenesis: 125 years after Pasteur.** *FEMS Microbiol Lett* 2006, **265:**1-10.

122. Weber DJ, Wolfson JS, Swartz MN, Hooper DC: **Pasteurella multocida infections. Report of 34 cases and review of the literature.** *Medicine (Baltimore)* 1984, **63:**133-154.

123. Lee DG, Urbach JM, Wu G, Liberati NT, Feinbaum RL, Miyata S, Diggins LT, He J, Saucier M, Deziel E, et al: **Genomic analysis reveals that Pseudomonas aeruginosa virulence is combinatorial.** *Genome Biol* 2006, **7:**R90.

124. Stover CK, Pham XQ, Erwin AL, Mizoguchi SD, Warrener P, Hickey MJ, Brinkman FS, Hufnagle WO, Kowalik DJ, Lagrou M, et al: **Complete genome sequence of Pseudomonas aeruginosa PAO1, an opportunistic pathogen.** *Nature* 2000, **406:**959-964.

125. Comer JA, Tzianabos T, Flynn C, Vlahov D, Childs JE: **Serologic evidence of rickettsialpox (Rickettsia akari) infection among intravenous drug users in inner-city Baltimore, Maryland.** *Am J Trop Med Hyg* 1999, **60:**894-898.

126. Radulovic S, Feng HM, Morovic M, Djelalija B, Popov V, Crocquet-Valdes P, Walker DH: **Isolation of Rickettsia akari from a patient in a region where Mediterranean spotted fever is endemic.** *Clin Infect Dis* 1996, **22:**216-220.

127. Hudson BJ, McPetrie R, Kitchener-Smith J, Eccles J: **Vesicular rash associated with infection due to Rickettsia australis.** *Clin Infect Dis* 1994, **18:**118-119.

128. Feng HM, Wen J, Walker DH: **Rickettsia australis infection: a murine model of a highly invasive vasculopathic rickettsiosis.** *Am J Pathol* 1993, **142:**1471-1482.

129. George F, Brouqui P, Boffa MC, Mutin M, Drancourt M, Brisson C, Raoult D, Sampol J: **Demonstration of Rickettsia conorii-induced endothelial injury in vivo by measuring circulating endothelial cells, thrombomodulin, and von Willebrand factor in patients with Mediterranean spotted fever.** *Blood* 1993, **82:**2109-2116.

130. Gudima OS: **Quantitative study on the reproduction of virulent and vaccine Rickettsia prow azeki strains in cells of different origin.** *Acta Virol* 1979, **23:**421-427.

131. Walker TS, Winkler HH: **Penetration of cultured mouse fibroblasts (L cells) by Rickettsia prowazeki.** *Infect Immun* 1978, **22:**200-208.

132. Clifton DR, Goss RA, Sahni SK, van Antwerp D, Baggs RB, Marder VJ, Silverman DJ, Sporn LA: **NF-kappa B-dependent inhibition of apoptosis is essential for host cellsurvival during Rickettsia rickettsii infection.** *Proc Natl Acad Sci U S A* 1998, **95:**4646-4651.

133. Niebylski ML, Peacock MG, Schwan TG: **Lethal effect of Rickettsia rickettsii on its tick vector (Dermacentor andersoni).** *Appl Environ Microbiol* 1999, **65:**773-778.

134. Fournier PE, Gouriet F, Brouqui P, Lucht F, Raoult D: **Lymphangitis-associated rickettsiosis, a new rickettsiosis caused by Rickettsia sibirica mongolotimonae: seven new cases and review of the literature.** *Clin Infect Dis* 2005, **40:**1435-1444.

135. Lewin MR, Bouyer DH, Walker DH, Musher DM: **Rickettsia sibirica infection in members of scientific expeditions to northern Asia.** *Lancet* 2003, **362:**1201-1202.

136. Labruna MB, Mattar S, Nava S, Bermudez S, Venzal JM, Dolz G, Abarca K, Romero L, de Sousa R, Oteo J, Zavala-Castro J: **Rickettsioses in Latin America, Caribbean, Spain and Portugal.** *Rev Mvz Cordoba* 2011, **16:**2435-2457.

137. Walker DH, Parks FM, Betz TG, Taylor JP, Muehlberger JW: **Histopathology and immunohistologic demonstration of the distribution of Rickettsia typhi in fatal murine typhus.** *Am J Clin Pathol* 1989, **91:**720-724.

138. Henry KM, Jiang J, Rozmajzl PJ, Azad AF, Macaluso KR, Richards AL: **Development of quantitative real-time PCR assays to detect Rickettsia typhi and Rickettsia felis, the causative agents of murine typhus and flea-borne spotted fever.** *Mol Cell Probes* 2007, **21:**17-23.

139. Michels F, Colaert J, Gheysen F, Scheerlinck T: **Late prosthetic joint infection due to Rothia mucilaginosa.** *Acta Orthop Belg* 2007, **73:**263-267.

140. Fanourgiakis P, Georgala A, Vekemans M, Daneau D, Heymans C, Aoun M: **Bacteremia due to Stomatococcus mucilaginosus in neutropenic patients in the setting of a cancer institute.** *Clin Microbiol Infect* 2003, **9:**1068-1072.

141. Baumler AJ, Tsolis RM, Ficht TA, Adams LG: **Evolution of host adaptation in Salmonella enterica.** *Infect Immun* 1998, **66:**4579-4587.

142. Chiu CH, Tang P, Chu C, Hu S, Bao Q, Yu J, Chou YY, Wang HS, Lee YS: **The genome sequence of Salmonella enterica serovar Choleraesuis, a highly invasive and resistant zoonotic pathogen.** *Nucleic Acids Res* 2005, **33:**1690-1698.

143. Glynn MK, Bopp C, Dewitt W, Dabney P, Mokhtar M, Angulo FJ: **Emergence of multidrug-resistant Salmonella enterica serotype typhimurium DT104 infections in the United States.** *N Engl J Med* 1998, **338:**1333-1338.

144. Hyma KE, Lacher DW, Nelson AM, Bumbaugh AC, Janda JM, Strockbine NA, Young VB, Whittam TS: **Evolutionary genetics of a new pathogenic Escherichia species: Escherichia albertii and related Shigella boydii strains.** *J Bacteriol* 2005, **187:**619-628.

145. Gross RJ, Threlfall EJ, Ward LR, Rowe B: **Drug resistance in Shigella dysenteriae, S flexneri and S boydii in England and Wales: increasing incidence of resistance to trimethoprim.** *Br Med J (Clin Res Ed)* 1984, **288:**784-786.

146. Kolavic SA, Kimura A, Simons SL, Slutsker L, Barth S, Haley CE: **An outbreak of Shigella dysenteriae type 2 among laboratory workers due to intentional food contamination.** *JAMA* 1997, **278:**396-398.

147. Lindberg AA, Brown JE, Stromberg N, Westling-Ryd M, Schultz JE, Karlsson KA: **Identification of the carbohydrate receptor for Shiga toxin produced by Shigella dysenteriae type 1.** *J Biol Chem* 1987, **262:**1779-1785.

148. Sansonetti PJ, Kopecko DJ, Formal SB: **Involvement of a plasmid in the invasive ability of Shigella flexneri.** *Infect Immun* 1982, **35:**852-860.

149. Sansonetti PJ, Phalipon A, Arondel J, Thirumalai K, Banerjee S, Akira S, Takeda K, Zychlinsky A: **Caspase-1 activation of IL-1 beta and IL-18 are essential for Shigella flexneri-induced inflammation.** *Immunity* 2000, **12:**581-590.

150. Sansonetti PJ, Kopecko DJ, Formal SB: **Shigella sonnei plasmids: evidence that a large plasmid is necessary for virulence.** *Infect Immun* 1981, **34:**75-83.

151. Gaastra W, Boot R, Ho HT, Lipman LJ: **Rat bite fever.** *Veterinary microbiology* 2009, **133:**211-228.

152. Dendle C, Woolley IJ, Korman TM: **Rat-bite fever septic arthritis: illustrative case and literature review.** *Eur J Clin Microbiol Infect Dis* 2006, **25:**791-797.

153. Holden MT, Feil EJ, Lindsay JA, Peacock SJ, Day NP, Enright MC, Foster TJ, Moore CE, Hurst L, Atkin R, et al: **Complete genomes of two clinical Staphylococcus aureus strains: evidence for the rapid evolution of virulence and drug resistance.** *Proc Natl Acad Sci U S A* 2004, **101:**9786-9791.

154. Lowy FD: **Staphylococcus aureus infections.** *N Engl J Med* 1998, **339:**520-532.

155. Zhang YQ, Ren SX, Li HL, Wang YX, Fu G, Yang J, Qin ZQ, Miao YG, Wang WY, Chen RS, et al: **Genome-based analysis of virulence genes in a non-biofilm-forming Staphylococcus epidermidis strain (ATCC 12228).** *Mol Microbiol* 2003, **49:**1577-1593.

156. Vuong C, Otto M: **Staphylococcus epidermidis infections.** *Microbes Infect* 2002, **4:**481-489.

157. Frank KL, Del Pozo JL, Patel R: **From clinical microbiology to infection pathogenesis: how daring to be different works for Staphylococcus lugdunensis.** *Clinical microbiology reviews* 2008, **21:**111-133.

158. Vandenesch F, Etienne J, Reverdy ME, Eykyn SJ: **Endocarditis due to Staphylococcus lugdunensis: report of 11 cases and review.** *Clin Infect Dis* 1993, **17:**871-876.

159. Kuroda M, Yamashita A, Hirakawa H, Kumano M, Morikawa K, Higashide M, Maruyama A, Inose Y, Matoba K, Toh H, et al: **Whole genome sequence of Staphylococcus saprophyticus reveals the pathogenesis of uncomplicated urinary tract infection.** *Proc Natl Acad Sci U S A* 2005, **102:**13272-13277.

160. Jordan PA, Iravani A, Richard GA, Baer H: **Urinary tract infection caused by Staphylococcus saprophyticus.** *J Infect Dis* 1980, **142:**510-515.

161. Elliott SP: **Rat bite fever and Streptobacillus moniliformis.** *Clinical microbiology reviews* 2007, **20:**13-22.

162. Rupp ME: **Streptobacillus moniliformis endocarditis: case report and review.** *Clin Infect Dis* 1992, **14:**769-772.

163. Glaser P, Rusniok C, Buchrieser C, Chevalier F, Frangeul L, Msadek T, Zouine M, Couve E, Lalioui L, Poyart C, et al: **Genome sequence of Streptococcus agalactiae, a pathogen causing invasive neonatal disease.** *Mol Microbiol* 2002, **45:**1499-1513.

164. Tettelin H, Masignani V, Cieslewicz MJ, Donati C, Medini D, Ward NL, Angiuoli SV, Crabtree J, Jones AL, Durkin AS, et al: **Genome analysis of multiple pathogenic isolates of Streptococcus agalactiae: implications for the microbial "pan-genome".** *Proc Natl Acad Sci U S A* 2005, **102:**13950-13955.

165. Tettelin H, Nelson KE, Paulsen IT, Eisen JA, Read TD, Peterson S, Heidelberg J, DeBoy RT, Haft DH, Dodson RJ, et al: **Complete genome sequence of a virulent isolate of Streptococcus pneumoniae.** *Science* 2001, **293:**498-506.

166. Whitney CG, Farley MM, Hadler J, Harrison LH, Lexau C, Reingold A, Lefkowitz L, Cieslak PR, Cetron M, Zell ER, et al: **Increasing prevalence of multidrug-resistant Streptococcus pneumoniae in the United States.** *N Engl J Med* 2000, **343:**1917-1924.

167. Cone LA, Woodard DR, Schlievert PM, Tomory GS: **Clinical and Bacteriological Observations of a Toxic Shock-Like Syndrome Due to Streptococcus-Pyogenes.** *New Engl J Med* 1987, **317:**146-149.

168. Holden MT, Scott A, Cherevach I, Chillingworth T, Churcher C, Cronin A, Dowd L, Feltwell T, Hamlin N, Holroyd S, et al: **Complete genome of acute rheumatic fever-associated serotype M5 Streptococcus pyogenes strain manfredo.** *J Bacteriol* 2007, **189:**1473-1477.

169. Lun ZR, Wang QP, Chen XG, Li AX, Zhu XQ: **Streptococcus suis: an emerging zoonotic pathogen.** *Lancet Infect Dis* 2007, **7:**201-209.

170. Arends JP, Zanen HC: **Meningitis caused by Streptococcus suis in humans.** *Rev Infect Dis* 1988, **10:**131-137.

171. Fraser CM, Norris SJ, Weinstock GM, White O, Sutton GG, Dodson R, Gwinn M, Hickey EK, Clayton R, Ketchum KA, et al: **Complete genome sequence of Treponema pallidum, the syphilis spirochete.** *Science* 1998, **281:**375-388.

172. Lukehart SA, Hook EW, 3rd, Baker-Zander SA, Collier AC, Critchlow CW, Handsfield HH: **Invasion of the central nervous system by Treponema pallidum: implications for diagnosis and treatment.** *Ann Intern Med* 1988, **109:**855-862.

173. Glass JI, Lefkowitz EJ, Glass JS, Heiner CR, Chen EY, Cassell GH: **The complete sequence of the mucosal pathogen Ureaplasma urealyticum.** *Nature* 2000, **407:**757-762.

174. Cassell GH, Waites KB, Watson HL, Crouse DT, Harasawa R: **Ureaplasma urealyticum intrauterine infection: role in prematurity and disease in newborns.** *Clinical microbiology reviews* 1993, **6:**69-87.

175. Howard-Jones N: **Robert Koch and the cholera vibrio: a centenary.** *Br Med J (Clin Res Ed)* 1984, **288:**379-381.

176. Heidelberg JF, Eisen JA, Nelson WC, Clayton RA, Gwinn ML, Dodson RJ, Haft DH, Hickey EK, Peterson JD, Umayam L, et al: **DNA sequence of both chromosomes of the cholera pathogen Vibrio cholerae.** *Nature* 2000, **406:**477-483.

177. Faruque SM, Albert MJ, Mekalanos JJ: **Epidemiology, genetics, and ecology of toxigenic Vibrio cholerae.** *Microbiol Mol Biol Rev* 1998, **62:**1301-1314.

178. Makino K, Oshima K, Kurokawa K, Yokoyama K, Uda T, Tagomori K, Iijima Y, Najima M, Nakano M, Yamashita A, et al: **Genome sequence of Vibrio parahaemolyticus: a pathogenic mechanism distinct from that of V cholerae.** *Lancet* 2003, **361:**743-749.

179. Daniels NA, MacKinnon L, Bishop R, Altekruse S, Ray B, Hammond RM, Thompson S, Wilson S, Bean NH, Griffin PM, Slutsker L: **Vibrio parahaemolyticus infections in the United States, 1973-1998.** *J Infect Dis* 2000, **181:**1661-1666.

180. Strom MS, Paranjpye RN: **Epidemiology and pathogenesis of Vibrio vulnificus.** *Microbes Infect* 2000, **2:**177-188.

181. Tacket CO, Brenner F, Blake PA: **Clinical features and an epidemiological study of Vibrio vulnificus infections.** *J Infect Dis* 1984, **149:**558-561.

182. Bottone EJ: **Yersinia enterocolitica: overview and epidemiologic correlates.** *Microbes Infect* 1999, **1:**323-333.

183. Portnoy DA, Moseley SL, Falkow S: **Characterization of plasmids and plasmid-associated determinants of Yersinia enterocolitica pathogenesis.** *Infect Immun* 1981, **31:**775-782.

184. Perry RD, Fetherston JD: **Yersinia pestis--etiologic agent of plague.** *Clinical microbiology reviews* 1997, **10:**35-66.

185. Eppinger M, Rosovitz MJ, Fricke WF, Rasko DA, Kokorina G, Fayolle C, Lindler LE, Carniel E, Ravel J: **The complete genome sequence of Yersinia pseudotuberculosis IP31758, the causative agent of Far East scarlet-like fever.** *PLoS Genet* 2007, **3:**e142.

186. El-Maraghi NR, Mair NS: **The histopathology of enteric infection with Yersinia pseudotuberculosis.** *Am J Clin Pathol* 1979, **71:**631-639.
